# Maternal Diet Determines Milk Microbiome Composition and Offspring Gut Colonization in Wistar Rats

**DOI:** 10.3390/nu15204322

**Published:** 2023-10-10

**Authors:** Paula Martínez-Oca, Claudio Alba, Alicia Sánchez-Roncero, Tamara Fernández-Marcelo, María Ángeles Martín, Fernando Escrivá, Juan Miguel Rodríguez, Carmen Álvarez, Elisa Fernández-Millán

**Affiliations:** 1 Instituto de Investigación en Ciencias de la Alimentación (CIAL), Campus de Excelencia Científica, Consejo Superior de Investigaciones Científicas-Universidad Autónoma de Madrid (CSIC-UAM), 28049 Madrid, Spain; paula.martinez.oca@csic.es; 2 Department of Nutrition and Food Science, Faculty of Veterinary Sciences, University Complutense of Madrid, 28040 Madrid, Spain; claudioalbarubio@gmail.com (C.A.); jmrodrig@ucm.es (J.M.R.); 3 Department of Biochemistry and Molecular Biology, Faculty of Pharmacy, Complutense University of Madrid, 28040 Madrid, Spain; alicis06@ucm.es (A.S.-R.); fescriva@ucm.es (F.E.); calvarez@ucm.es (C.Á.); 4 Centro de Investigación Biomédica en Red (CIBERDEM), ISCIII, 28029 Madrid, Spain; tafernan@ucm.es (T.F.-M.); amartina@ictan.csic.es (M.Á.M.); 5 Department of Metabolism and Nutrition, Institute of Food Science and Technology and Nutrition (ICTAN), Consejo Superior de Investigaciones Científicas (CSIC), 28040 Madrid, Spain

**Keywords:** milk microbiome, metabolic programming, gut colonization, food restriction, lactation

## Abstract

Mother’s milk contains a unique microbiome that plays a relevant role in offspring health. We hypothesize that maternal malnutrition during lactation might impact the microbial composition of milk and affect adequate offspring gut colonization, increasing the risk for later onset diseases. Then, Wistar rats were fed ad libitum (Control, C) food restriction (Undernourished, U) during gestation and lactation. After birth, offspring feces and milk stomach content were collected at lactating day (L)4, L14 and L18. The V3–V4 region of the bacterial 16S rRNA gene was sequenced to characterize bacterial communities. An analysis of beta diversity revealed significant disparities in microbial composition between groups of diet at L4 and L18 in both milk, and fecal samples. In total, 24 phyla were identified in milk and 18 were identified in feces, with Firmicutes, Proteobacteria, Actinobacteroidota and Bacteroidota collectively representing 96.1% and 97.4% of those identified, respectively. A higher abundance of *Pasteurellaceae* and *Porphyromonas* at L4, and of *Gemella* and *Enterococcus* at L18 were registered in milk samples from the U group. *Lactobacillus* was also significantly more abundant in fecal samples of the U group at L4. These microbial changes compromised the number and variety of milk–feces or feces–feces bacterial correlations. Moreover, increased offspring gut permeability and an altered expression of goblet cell markers TFF3 and KLF3 were observed in U pups. Our results suggest that altered microbial communication between mother and offspring through breastfeeding may explain, in part, the detrimental consequences of maternal malnutrition on offspring programming.

## 1. Introduction

Early life adverse events play an important role in the etiology of many diseases. In particular, lactation is considered a critical programming window, being a sensitive period in which to establish neural connections, behavior responses and metabolic circuits [1,2]. According to this, altered maternal nutrition has been the most extensively studied programming challenge for offspring metabolic diseases, including obesity and type 2 diabetes (T2D) [3,4,5,6]. Under optimal conditions, maternal milk is regarded the best feeding source for neonates, providing all nutrient requirements to ensure adequate growth and maturation. However, a hypocaloric or low-protein diet during lactation can have a negative impact on breast milk production [7], as well as on its content of nutrients [8,9], and the milk intake by young pups [8,9,10], exerting negative effects on their development. Other studies in rodents, under moderate or mild caloric restriction during lactation (a 20% restriction of ad libitum feeding), via metabolomic analysis, identified changes in 29 metabolites related to various metabolic pathways [11]. The mechanisms by which breast milk programs infants to a low or high risk of becoming obese or glucose intolerant at adulthood are not fully defined but this metabolic dysfunction has been associated with altered insulin sensitivity [12], hepatic glycogen metabolism [13], a disrupted leptin production profile [14] and damaged hypothalamic circuits [14,15]. Moreover, we and others have also described that maternal food restriction leads to altered intestinal barrier maturation and the altered function of the offspring [4,16,17], promoting the development of local or extra-intestinal inflammatory events [16].

It is worth noting that maternal milk composition refers not only to essential nutrients (lipids, carbohydrates, proteins, vitamins or minerals) but also to other bioactive factors, such as hormones (insulin or leptin), cytokines, immunoglobulins, and numerous types of oligosaccharides, among others [2]. For a long time thought to be a sterile body fluid, nowadays it is widely accepted that breast milk has also its own microbiota, able to influence newborns’ immune systems [18]. The origin of this microbiota is still uncertain. One hypothesis suggests that, through the process of breastfeeding, skin bacteria may be transferred to breast milk or directly to the person lactating. Another hypothesis suggests that a significant milk reflux from the infant’s oral cavity through the nipple into the mammary gland may also occur. Finally, an entero-mammary trafficking model has been proposed as well [19]. However, regardless of the route via which bacteria enter the mammary gland, breast milk constitutes the key source of microbes for neonates’ gut colonization. Human studies have revealed that the milk of healthy women contains a great diversity of bacteria, but with a core of genera including *Streptococcus* and *Staphylococcus*, followed by *Lactobacillus*, *Bifidobacterium* and *Enterococcus* [20,21]. Interestingly, the genera *Streptococcus* and *Staphylococcus*, along with *Bifidobacterium*, have been described as pioneer anaerobic bacteria colonizing the gut niche in the first days of life [20,22], demonstrating vertical mother-to-child bacterial transfer through breastfeeding [20]. The breast milk microbiome can vary depending on the phase of lactation we are in, the maternal metabolic status or her body mass index as well as the weight gain she experienced during pregnancy or the type of delivery [23,24]. In this regard, it has been seen that obese mothers have a different and less diverse milk microbial composition than that of normal-weight mothers [24], which is characterized by low levels of *Bifidobacterium* but high levels of *Staphylococcus* [25]. In agreement, lower levels of *Bifidobacterium* have been reported in feces from obese children compared to those of normal weight for the same age [26]. Moreover, animal studies, including ours, have also demonstrated that the diet restriction of mothers impacts milk microbial diversity [27] and causes a shift in the composition of offspring gut microbiota [16,27], which precedes the onset of metabolic syndrome [4,16], suggesting a causal relationship. Thus, given the significant association between microbiome composition and metabolic disease outcomes, the maternal dietary influence on offspring disease development may be partially explained by the dietary modulation of milk microbial components impacting the offspring gut’s primary colonization. However, to our knowledge, no studies have performed a detailed characterization of the milk microbiome during lactation in parallel to the study of the temporal progress of gut microbial colonization. Therefore, the primary purpose of this study is to determine the influence of maternal diet (a 65% food restriction of Wistar rats) on the milk microbiome and whether or not variations in the milk microbiome are related to variations in the neonate fecal microbiome. To this end, here we analyzed longitudinal maternal milk and offspring fecal samples via metataxonomic sequencing, from a few days after birth to the time close weaning.

## 2. Materials and Methods

### 2.1. Animals and Diets

The Institutional Animal Care and Use Committee of the Community of Madrid approved all methods for this study (PROEX 349/15 and PROEX 215.8/21). The investigators were accredited, and the animal facilities were approved by the General Administration of Agriculture and Livestock of the Autonomous Government of Madrid (Spain). Ten-week-old Wistar rats, obtained from Janvier Labs (France), were housed under specific pathogen-free and standard laboratory conditions (12:12 h light cycle; 21 °C) with ad lib access to water and a normal chow diet (2.9 kcal/g; 12% fat, 60% carbohydrates and 28% protein; A04, SAFE). After 10 days of habituation, females were housed with males overnight, and two weeks after mating, pregnant rats were randomly assigned to one of the two treatment groups. The first group was fed ad lib with the mentioned standard chow diet (control (C), *n* = 6), and the second group was 65% food restricted during the last third of gestation and postnatally (undernourished (U), *n* = 6), as previously described [16]. To reduce the effect of litter size on postnatal growth and milk intake, litters were made uniform at birth, to eight pups per nursing dam. Food restriction was maintained in the mothers during lactation, and pups were sacrificed via decapitation at 4, 14 and 18 days of life (L4, L14 and L18) to collect the different types of samples. The weight gain of pups was monitored throughout lactation and the body and gastrointestinal system length (from the pylorus to the anus) were measured to determine the nutritional restriction effects on the organ sizes. This animal model is designed to monitor lactation until L18, which is 5 days before weaning, to prevent the pups from beginning to ingest the mother’s chow diet by themselves and thus ensure there was no mixed diet.

### 2.2. Sample Collection

Milk collection for its microbiological analysis was always conducted at the same time (9:00 a.m.) and under sterile conditions. Although rat dams produce copious amounts of milk relative that produced by smaller rodents, the impact of long-term food restriction, from mid-gestation through to lactation, significantly limited the availability of milk from mothers’ breasts. Prior research on rodents has suggested that the composition of stomachal milk is similar to that of breastmilk [28], representing vertical transmission between mother and child. Thus, after sacrifice, the pups’ stomachs were dissected, and the contents were kept at 4 °C and immediately processed. Likewise, fecal colonic content was collected aseptically from pups into a sterile tube, 0.5 mL of TE50 buffer (10 mM Tris-HCl and 50 mM EDTA, pH 8) was added, the mixture was vortexed until it was homogeneous, and then it was frozen at −80 °C until DNA extraction and microbial studies were performed. For gene expression analysis, the distal colon was opened longitudinally and cleansed with saline solution. For histological studies, the colon was dissected and, maintaining its cylindrical structure and fecal content, immersed in Carnoy fixative solution.

### 2.3. Analytical Determinations

Before sacrifice via decapitation, the neonates were weighed and then blood samples were collected; their serum was separated and stored frozen at −20 °C until analysis. Serum insulin and glucagon levels were determined with a specific rat insulin and glucagon radioimmunoassay kit (LINCO Research, Inc., Merck Millipore, St. Louis, MI, USA), in accordance with the manufacturer’s protocol. A sensitivity of 0.1 ng/mL and of 20 pg/mL was achieved with overnight equilibrium using 100 μL of the serum samples, respectively. The coefficients of variation within and between assays were 10% for insulin and 4.0 and 7.3% for glucagon, respectively. Serum glucose and lipid parameters (total cholesterol and triglycerides) were determined via the enzymatic colorimetric glucose oxidase/peroxidase method (Biosystems, Barcelona, Spain).

### 2.4. DNA Extraction

#### 2.4.1. Milk

The samples underwent centrifugation at 4 °C for 15 min at a relative centrifugal force of 11,000× *g*. DNA was then extracted from the resultant pellets using QIAamp DNA Stool Mini Kit (Qiagen, Hilden, Germany), in accordance with the manufacturer’s guidelines, albeit with previously documented modifications [29]. The extracted DNA was eluted in 20 μL of nuclease-free water and subsequently stored at −20 °C pending additional analyses. The DNA concentration was estimated utilizing NanoDrop ND-1000 UV-Vis Spectrophotometer (NanoDrop Technologies, Wilmington, DE, USA). The DNA extraction and purification protocol delineated above was also applied to two negative control blanks, which did not contain any sample material.

#### 2.4.2. Feces

The frozen fecal samples were rapidly thawed using a dry heat block set at 37 °C, followed by vortexing to achieve re-homogenization. DNA was extracted utilizing QIAamp Fast DNA Stool Mini Kit (Qiagen, Germantown, MD, USA) in conjunction with 0.1 mm diameter zirconia/silica beads (BioSpec Products, Inc., Bartlesville, OK, USA) and a FastPrep FP120A-115 homogenizer (Qbiogene, Carlsbad, CA, USA). During each round of DNA extraction, 500 μL of TE50 buffer from the same aliquot was used in the sample and 500 μL of nuclease-free water (Ambion, Waltham, MA, USA) was used for negative controls. The extracted DNA samples were eluted in 20 μL of ATE buffer, as provided in the extraction kit, and stored at −80 °C until further analysis.

### 2.5. DNA Quality, Quantification, and Sequencing

DNA quality and quantification were assessed as previously described [20]. Briefly, the concentration of the DNA samples was determined using the Quant-iT PicoGreen reagent (Thermo Fisher Scientific, Inc., Waltham, MA, USA). Amplification targeted the V3–V4 region of the 16S rRNA gene. Both negative and positive control PCR products were subjected to agarose gel electrophoresis at 80 V for 30 min using 1% agarose gel and TAE (Tris-Acetate-EDTA) buffer. The gel was stained with GelRed™ (10X, Biotium, Fremont, CA, USA) and visualized using UltraCam Digital Imaging System (Bio-Rad, Hercules, CA, USA). Amplicon quality was assessed with a QIAxcel DNA Screening cartridge (Qiagen, Hilden, Germany) in accordance with the manufacturer’s guidelines. High-resolution capillary electrophoresis on a QIAxcel Advanced System (Qiagen) was employed for visualization. Samples demonstrating a peak at the desired amplicon size and minimal primer dimer formation were deemed to be of sufficient quality.

Subsequent to primary PCR amplification (the resulting amplicons being approximately 450 bp in length), the individual amplicon libraries were characterized using Bioanalyzer 2100 (Agilent Technologies, Palo Alto, CA, USA). The libraries were then pooled in equimolar concentrations, followed by purification and quantification. The precise concentration of the pooled sample was confirmed via real-time PCR (Kapa Biosystems, Wilmington, MA, USA). Finally, the pooled DNA samples underwent sequencing on an Illumina MiSeq instrument, employing 2 × 300 bp paired-end read sequencing at the Genomics Unit of Parque Científico de Madrid, Spain.

In accordance with manufacturer specifications, sequences underwent demultiplexing facilitated by Illumina’s software platform (v. 2.6.2.3). Subsequent bioinformatic analysis was conducted utilizing Quantitative Insights into Microbial Ecology 2 (QIIME 2, version 2022.2) in conjunction with R Statistical Computing Environment (version 3.5.1; URL: https://www.r-project.org/ accessed on 13 January 2023). For the optimization of sequence integrity, the DADA2 algorithm was employed under specific parameters: forward reads were truncated post-position 295 while excising the initial 15 nucleotides; conversely, reverse reads were truncated at position 258 with the preliminary 7 nucleotides eliminated. This procedure was implemented to obviate positions demonstrating a median nucleotide quality score inferior to Q20. Taxonomic affiliation for individual amplicon sequence variants (ASVs) was ascertained through the q2 feature classifier, utilizing a naive Bayes taxonomic classification schema against the SILVA 138.1 reference database. Lastly, potential contaminants were identified, visualized and purged from the dataset via the decontam package (version 1.2.1), incorporating data from two negative control samples for rigorous verification.

### 2.6. Metataxonomic Analysis of Bacterial Microbiota

A matrix detailing ASVs specific to each respective sample was systematically generated. The subsequent normalization of bacterial taxa abundances was performed utilizing the total sum scaling (TSS) method, where in each discrete ASV count was divided by the cumulative library size, thereby rendering the relative proportion of individual ASV counts per sample. For the examination of alpha diversity, both Shannon and Richness diversity indices were employed, facilitated through the vegan package within R Statistical Computing Environment (Version 2.5.6).

To provide a comprehensive assessment of community structure, beta diversity was analyzed using both Bray–Curtis and binary Jaccard dissimilarity metrics. These metrics serve to quantify the compositional dissimilarity between microbial communities across samples. All computations and statistical manipulations were executed within the R framework, ensuring methodological consistency and analytical rigor.

### 2.7. Immunofluorescence Staining

The immunofluorescence staining for tight junction protein zonula occludens-1 (ZO-1) was performed on colonic sections as previously described [16]. Briefly, after being deparaffinized and rehydrated, the antigens were retrieved with sodium citrate buffer (10 mM Na_3_C_6_H_5_O_7_ and Tween-20 0.05%, pH 6) at 97 °C for 30 min. Non-specific background was blocked via incubation with 2% BSA, 1% NGS and 0.2% Triton X-100 in PBS for 1 h at room temperature. Sections were incubated overnight at 4 °C with rabbit anti-ZO-1 (1:50, ThermoFisher Scientific, Inc., Waltham, MA, USA) and then probed in the dark with a 1:100 dilution of Alexa Fluor 488-conjugated goat anti-rabbit IgG antibody and a 1:1000 dilution of 10 mg/mL of DAPI solution (Thermo Fisher Scientific, Waltham, MA, USA) for 1 h at room temperature. Finally, the sections were covered with ProLong Gold Antifade Mountant (Invitrogen, Thermo Fisher Scientific, Inc., Waltham, MA, USA). Negative control slides were obtained by incubating them without the primary antibody but with a secondary Alexa Fluor antibody. For quantification, images were captured using a Leica SP-2 AOBS confocal microscope at 630× magnification. At least two non-adjacent sections of colon per rat were stained and four images per section were used. Two independent experiments were conducted, with 2–3 rats per group in each experiment. To measure the intensity of the stained area, Fiji (ImageJ software v. 2.14.0) was employed [30].

### 2.8. Inner Mucus Layer Thickness Analysis

Samples from the distal colon were harvested and immediately preserved in Carnoy’s fixative (dry methanol: chloroform: glacial acetic acid in the ratio 60:30:10) as described earlier [16]. After 4 h in Carnoy’s solution, the colons were washed in dry methanol and ethanol 100% for 1 h, cleaned with xylene and embedded in paraffin wax. Then, the Alcian blue pH 2.5 (AB) (AB-8GX, PanReac Applichem; Schiff’s reagent, VWR Chemicals) staining of 5 μm sections was performed. Only regions in which the mucus layer was sandwiched between the epithelium on one side and luminal content on the other were used for measurements of thickness. Care was taken to consider only those regions that represented regular thickness; 6 to 8 measurements were taken per image, averaged over the total usable area of the colon. Histological determinations were performed using a light microscope (Eclipse 80i, Nikon, Tokyo, Japan) connected to a digital camera (XCD-U100CR, Sony, Tokyo, Japan). Morphometric characteristics were analyzed using Histolab software v. 7.0 (Microvision Instruments, EVRY Cedex, France. Measurements were always single-blinded.

### 2.9. RNA Extraction and Quantitative RT-PCR

Total RNA was extracted from frozen colonic tissues using TRIzol Reagent (Invitrogen, Thermo Fisher Scientific, Inc., Waltham, MA, USA) and reverse transcribed using a high-capacity cDNA reverse transcription kit (Applied Biosystems, ThermoFisher Scientific, Inc., Waltham, MA, USA). Real-time quantitative PCR analyses were performed using forward and reverse primers (Table 1) to determine the relative abundance of *Muc2*, *Tff1*, *Tff3* and *Klf3* genes. The comparative threshold cycle method was used to calculate relative expression. The target gene values were normalized to the expression of the endogenous reference (18S) (Table 1).

### 2.10. Statical and Bioinformatic Analyses

The distribution of the data was initially assessed using the Shapiro–Wilk normality test. Data conforming to a normal distribution were expressed as the mean ± standard error of the mean (SEM) or the mean alongside the 95% confidence interval (95% CI). When data were not normally distributed, they were presented as the median coupled with the interquartile range (IQR). For the comparison of two groups, significance was assessed using a 2-tailed Student’s *t*-test for normally distributed data and Wilcoxon rank sum tests for non-parametric data, with a significance level set at *p* < 0.05 for all tests. For comparisons involving more than two groups, analysis of variance (ANOVA) was used for normally distributed data, whereas the Kruskal–Wallis test was employed for non-parametric data. Post hoc analyses included pairwise *t*-tests for ANOVA and Wilcoxon rank sum tests with continuity correction for the Kruskal–Wallis test.

Further specialized analyses included the evaluation of median relative abundances of dominant taxa using Kruskal–Wallis tests or Wilcoxon rank tests, followed by appropriate multiple comparison corrections such as the Bonferroni method. Alpha diversity was measured using the Shannon diversity index, which considers both the number and evenness of microbial species. Beta diversity was visualized through principal coordinates analysis (PCoA) based on distance matrices, with Bray–Curtis and binary Jaccard indices used for quantitative and qualitative analyses, respectively. PERMANOVA with 999 permutations was performed to reveal statistically significant differences in beta diversity (*p* < 0.05). Pearson rank correlation analyses were conducted to evaluate potential correlations between bacterial genera in milk and fecal samples, as well as within fecal samples alone. All statistical and bioinformatic analyses were conducted using R software, combining version 3.3.2 and version 4.0.3 (R-project, http://www.r-project.org, accessed on 13 January 2023), along with QIIME pipelines (v. 1.8.0).

## 3. Results

### 3.1. Effect of Nutritional Restriction on the Biochemical Profile of Offspring Rats during Lactation

Both nutritional restriction and the particular metabolic adaptations that experience the mother during gestation and lactation may contribute to the early programming of an offspring. In this context, maternal food restriction during the last third of pregnancy resulted in pups with a significant lower body weight at birth compared to that of C newborns, but without statistical differences in the litter size and only a slight tendency of increase in the male-to-female ratio (*p* = 0.09) (Table 2). Consistent with previous data [12], nutritional restricted lactating rats (U) showed a significant reduction in body weight gain compared to that of C animals, reaching a difference of almost a 50% at the end of the period (L18) (Table 3). Similarly, serum glucose, insulin and glucagon levels remained significantly lower throughout lactation in U animals. In the case of the serum lipid profile, there were no differences between the two groups (C and U) at any age. However, while cholesterol and triglycerides levels were high in both C and U pups at the beginning of lactation (L4), these levels decreased and became stable with increasing age (Table 3).

### 3.2. Effect of Maternal Diet on the Milk and Gut Microbiome of Offspring throughout Lactation

#### 3.2.1. Metataxonomic Analysis of the Milk Samples

**Figure 1:**
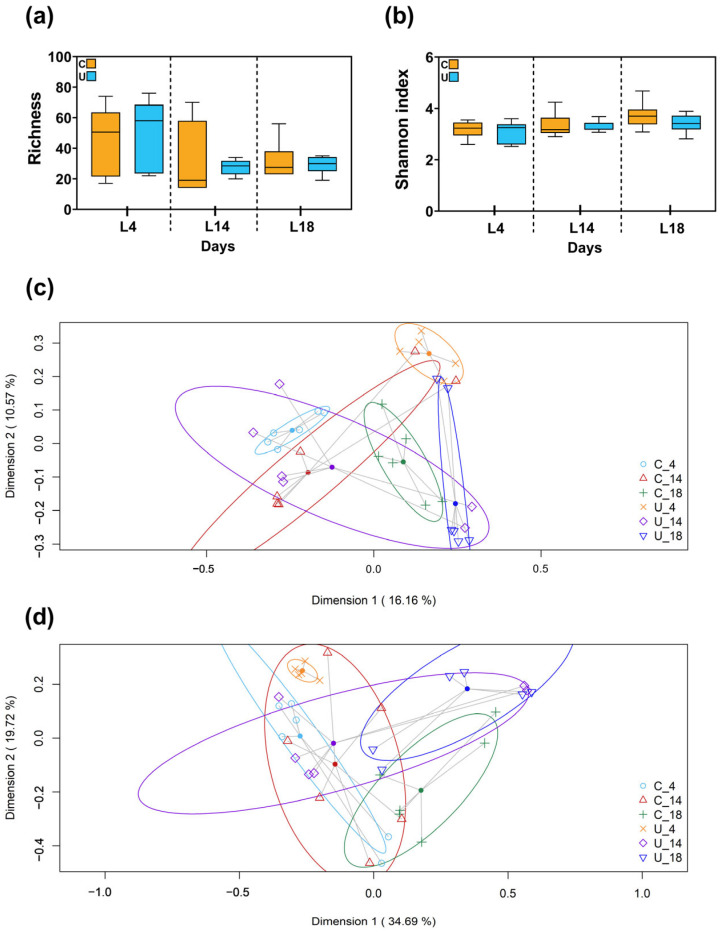
Microbial alpha diversity in milk samples expressed by (**a**) the number of observed genera (genera richness) and (**b**) the Shannon index. Box plots represent median and interquartile ranges together with maximum and minimum values. Kruskal–Wallis analysis was used to evaluate differences between groups (in terms of lactational age and type of diet). The Beta diversity analysis of milk samples was measured as (**c**) the presence/absence (Binnary Jaccard method) or (**d**) the relative abundance (Bray–Curtis similarity analysis) of the different species quantified in control groups (yellow: C4; orange: C14; red: C18) and undernourished groups (light blue: U4; cyan blue: U14; dark blue: U18). The value given on each axis label represents the percentage of the total variance explained by that axis. Permanova analysis was used to evaluate differences between groups (in terms of lactational age and type of diet). N = 5–6 per condition. C: control; U: undernourished. L: lactation.

The comparative metataxonomic analysis of microbial profiles in milk samples, stratified according to nutritional status (C vs. U) and days of lactation (L4, L14 and L18), was conducted utilizing 35 of the samples as delineated in Section 2.5. From these 35 milk samples, a cumulative total of 811,904 high-quality filtered sequences was obtained. The sequence count per sample oscillated between 12,569 and 33,787 (median [IQR]: 22,540 [18,872.5–28,013]), which could be clustered into 2586 distinct ASVs. The alpha diversity within these milk samples, irrespective of whether they were from C or U groups, exhibited comparable Richness and Shannon diversity indices across all time points of lactation (L4, L14 and L18), as depicted in Figure 1a,b.

An analysis of beta diversity was conducted to compare the C and U groups at each designated lactational time point. Two-dimensional principal coordinates analysis (2D-PCoA) utilizing Jaccard distance metrics provided a visual platform for assessing the extent of sample clustering based on both nutritional status and lactational stage. Notably, distinct clustering patterns emerged at day 4 and day 18 (Figure 1c), suggesting a significant divergence in microbial profiles between C and U cohorts at these sampling points. This observation was substantiated statistically via a subsequent PERMANOVA analysis based on Jaccard similarity metrics, which revealed significant differences in bacterial composition between the two nutritional groups (*p* = 0.009 and *p* = 0.014). Additionally, a separate PCoA plot generated using Bray–Curtis similarity indices, which were normalized relative to the abundance of distinct ASVs, demonstrated significant clustering predicated on nutritional status at L4 (*p* = 0.029), but not at L14 or L18 (*p* > 0.05) (Figure 1d).

**Figure 2:**
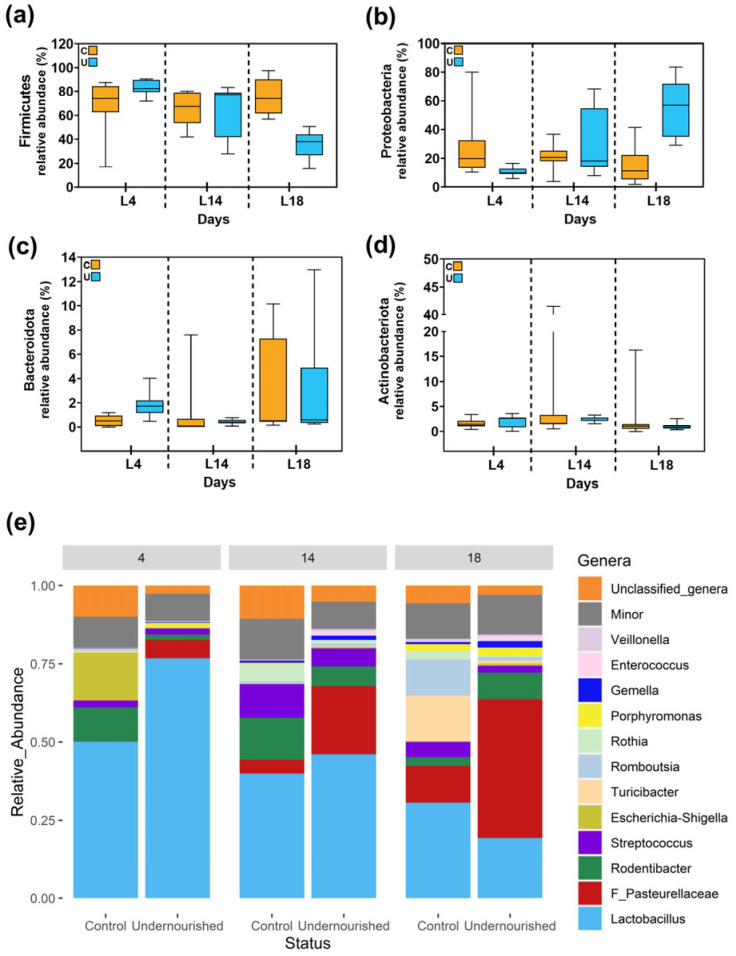
Comparison of the relative abundance of sequences belonging to main bacterial phyla (**a**) Firmicutes, (**b**) Proteobacteria, (**c**) Bacteroidota and (**d**) Actinobacteriota, and (**e**) the most abundant genera obtained via metataxonomic analysis in milk samples at lactating days L4, L14 and L18 from control (C) and undernourished (U) animals. Box plots represent median and interquartile ranges together with maximum and minimum values. Wilcoxon analysis was used to evaluate differences between groups in terms of type of diet. N = 5–6. The F preceding Pasteurelleceae in milk samples refers to (**e**) family taxon.

At the taxonomic level, discernible differences in bacterial relative abundances were observed related to diet type at specific time points. Taxa belonging to the Firmicutes and Proteobacteria phyla dominated in milk samples from both C and U animals. Other phyla such as Bacteroidota and Actinobacteroidota were also present but at a lower relative abundance. Although Firmicutes showed a tendency to decrease and Proteobacteria showed a tendency to increase their relative abundance in the U group at L18, no statistical significance was detected when compared to that of their C counterparts (Figure 2a–d). On the other hand, the relative abundance of sequences belonging to the family *Pasteurellaceae* was significantly higher in the samples from the U group (median [IQR] = 5.57 [3.33–8.2]) than in those from C animals (median [IQR] = < 0.01 [<0.01–<0.01]) at L4 (*p* = 0.0055). Similarly, the genera *Gemella* and *Enterococcus* manifested elevated relative abundances in the U samples at L18, with median [IQR] values of 0.94 [0.52–2.19] vs. 0.64 [0.41–0.85] (*p* = 0.007) and 0.08 [<0.01–2.61] vs. < 0.01 [<0.01–0.03] (*p* = 0.030), respectively. In contrast, the genus Romboutsia was more abundant in the C group at L18 (*p* = 0.015). Additionally, Porphyromonas was more prevalent in U samples at L4, with a median [IQR] of 0.95 [0.67–1.66] vs. 0.1 [0.01–0.25] (*p* = 0.024). No significant differences in the relative abundances of several other taxa, such as *Lactobacillus*, *Rodentibacter*, Unclassified_genera, *Streptococcus*, *Escherichia-Shigella*, *Turicibacter*, *Rothia* and *Veillonella*, were detected between C and U at any of the ages considered (Figure 2e and Table S1).

#### 3.2.2. Metataxonomic Analysis of the Fecal Samples

**Figure 3:**
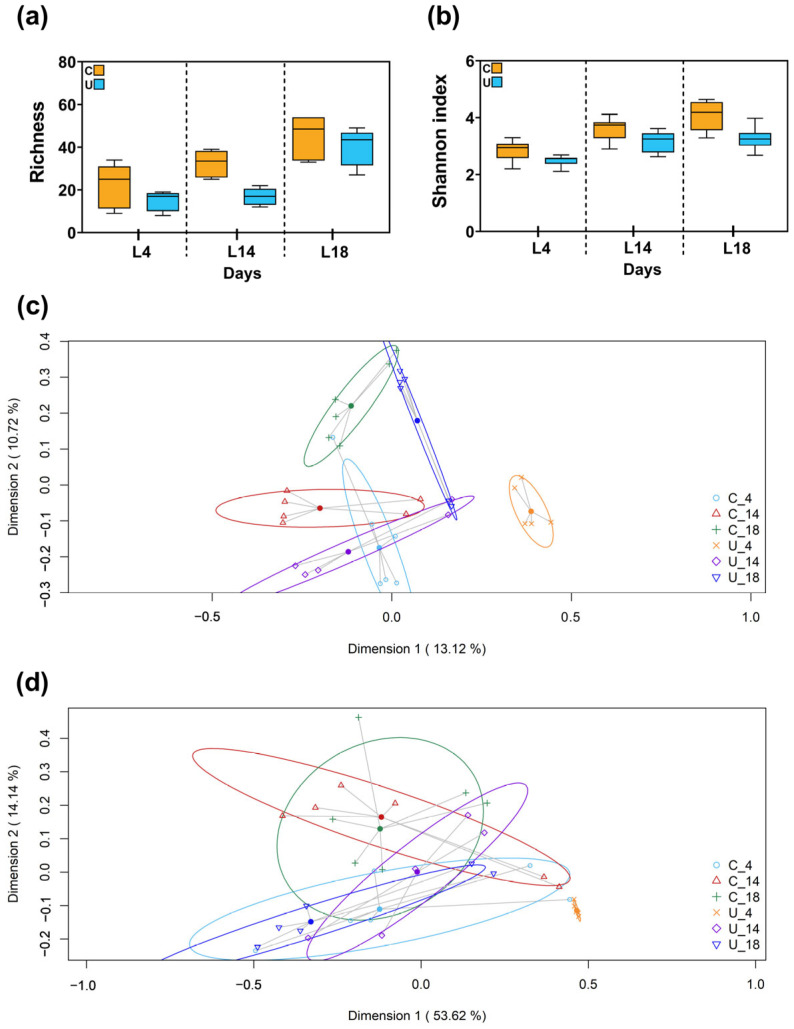
Microbial alpha diversity in feces samples expressed by (**a**) the number of the observed genera (genera richness) and (**b**) the Shannon index. Box plots represent median and interquartile ranges together with maximum and minimum values. Kruskal–Wallis analysis was used to evaluate differences between groups (in terms of lactational age and type of diet). The beta diversity analysis of milk samples was measured as (**c**) the presence/absence (Binnary Jaccard method) or (**d**) the relative abundance (Bray–Curtis similarity analysis) of the different species quantified in control groups (yellow: C4; orange: C14; red: C18) and undernourished groups (light blue: U4; cyan blue: U14; dark blue: U18). The value given on each axis label represents the percentage of the total variance explained by that axis. Permanova analysis was used to evaluate differences between groups (in terms of lactational age and type of diey). N = 5–6 per condition. C: control; U: undernourished. L: lactation.

In a subsequent phase of our investigation, we directed our analytical focus towards the fecal microbiota, utilizing the same categorical divisions based on nutritional status (C vs. U) and specific postnatal days (L4, L14 and L18). Of the initial 35 fecal samples, one was excluded from analysis due to the very low sequence count. Consequently, our study proceeded with a final collection of 34 fecal samples, from which we obtained a cumulative total of 915,417 high-quality filtered sequences. These sequences were distributed across samples with counts ranging from 15,750 to 40,935, with a median sequence count of 27,200 (interquartile range [IQR]: 21,193.25–31,677.5). Afterwards, these high-quality sequences were organized into 2586 distinct ASVs. In relation to alpha diversity, no statistically significant variations were observed between C and U groups, as evidenced by the Richness and Shannon indices across the three postnatal time frames (Figure 3a,b).

The analysis of beta diversity, with the two-dimensional principal coordinates analysis (2D-PCoA) employing Jaccard distance metrics offered a visual framework for exploring sample distribution. Notably, distinct clustering was found at postnatal days 4 (L4) and 18 (L18) (Figure 3c). These observations underwent statistical validation through a PERMANOVA analysis; employing Jaccard similarity metrics, the analysis revealed significant disparities in microbial composition between the two groups of diet at these specific time points (*p* = 0.009 for L4, *p* = 0.014 for L18). However, when employing Bray–Curtis similarity metrics for a parallel PCoA representation, no significant differences in clustering based on nutritional status were observed at any age studied (*p* > 0.05) (Figure 3d).

**Figure 4:**
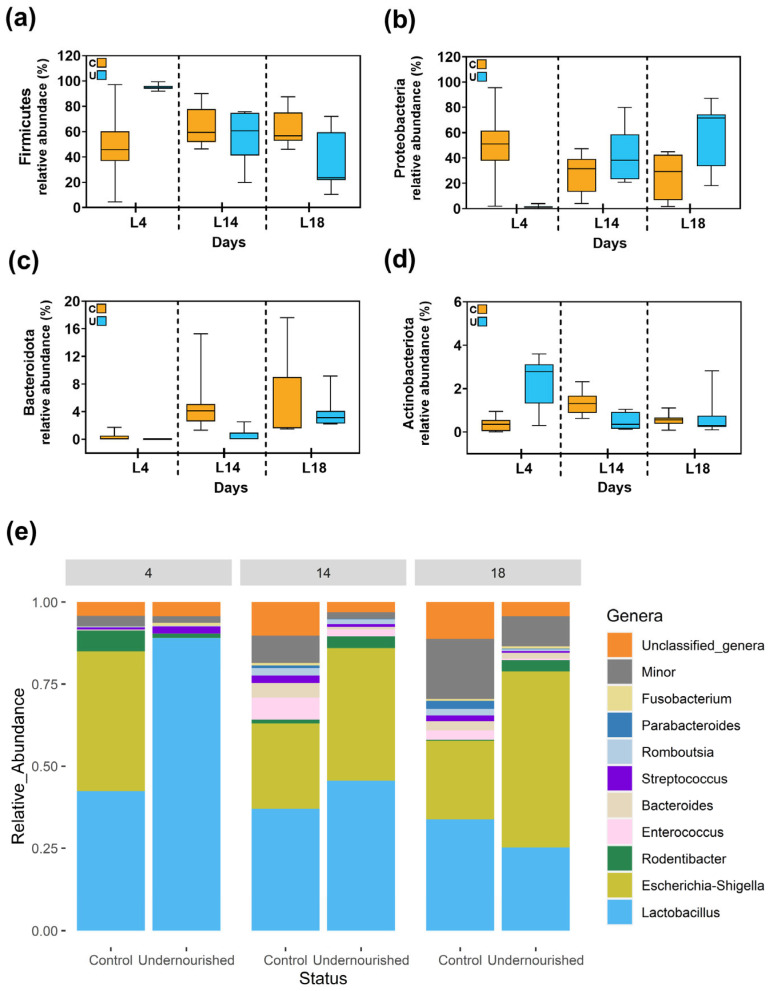
Comparison of the relative abundance of sequences belonging to main bacterial phyla (**a**) Firmicutes, (**b**) Proteobacteria, (**c**) Bacteroidota and (**d**) Actinobacteriota, and (**e**) the most abundant genera obtained via metataxonomic analysis in feces samples at lactating days L4, L14 and L18 from control (C) and undernourished (U) animals. Box plots represent median and interquartile ranges together with maximum and minimum values. Wilcoxon analysis was used to evaluate differences between groups in terms of type of diet. N = 5–6.

Subsequently, the taxonomic landscape of fecal samples revealed discernible differences in bacterial abundances between the C and U groups at specific postnatal time points. In total, 18 phyla were identified in fecal samples with Firmicutes, Proteobacteria, Bacteroidota and Actinobacteria representing 97.4% of those identified (Figure 4a–d). Notably, at the genus level, *Lactobacillus* exhibited a statistically significant overrepresentation in the U cohort on day L4, with median abundances of [IQR] = 88.57 [84.63–90.89] compared to those of [IQR] = 41.12 [35.85–49.84] in C animals (*p* = 0.0261). Conversely, the genus *Streptococcus* displayed increased relative abundances on days L14 and L18 in the C group, with median abundances of [IQR] = 2.43 [1.84–2.91] and 1.53 [0.85–2.34] compared to those of [IQR] = 0.75 [0.4–1.12] and 0.47 [0.35–0.67] in the group subjected to food restriction (*p* = 0.0261 for both days) (Figure 4e and Table S2). The study of the genera composition of fecal samples revealed substantial interindividual variation between the animals at L4 (Table S2) which implied that, despite *Escherichia-Shigella* being detected at a high relative abundance in 83.33% of C samples with a median abundance of [IQR] = 49.33 [12.24–59.37] compared to that of [IQR] < 0.01 [<0.01–<0.01] detected only in 1 sample from the U group, these microbial shifts did not reach statistical significance. Likewise, *Bacteroides* was detected in the 50% of C samples at L4 but not in any of the U group for the same time point. On the other hand, *Fusobacterium* was identified in the 100% samples from U pups at L4 whereas only two samples from C animals revealed the presence of this bacteria at L4. Other genera, including Unclassified_genera, *Rodentibacter*, *Enterococcus*, *Romboutsia* and *Parabacteroides* also did not show statistically significant differences in relative abundances between the nutritional groups across the time points examined (Figure 4e and Table S2).

### 3.3. Pearson Rank Correlation Analysis between Predominant Genera in Milk and Offspring Feces

**Figure 5:**
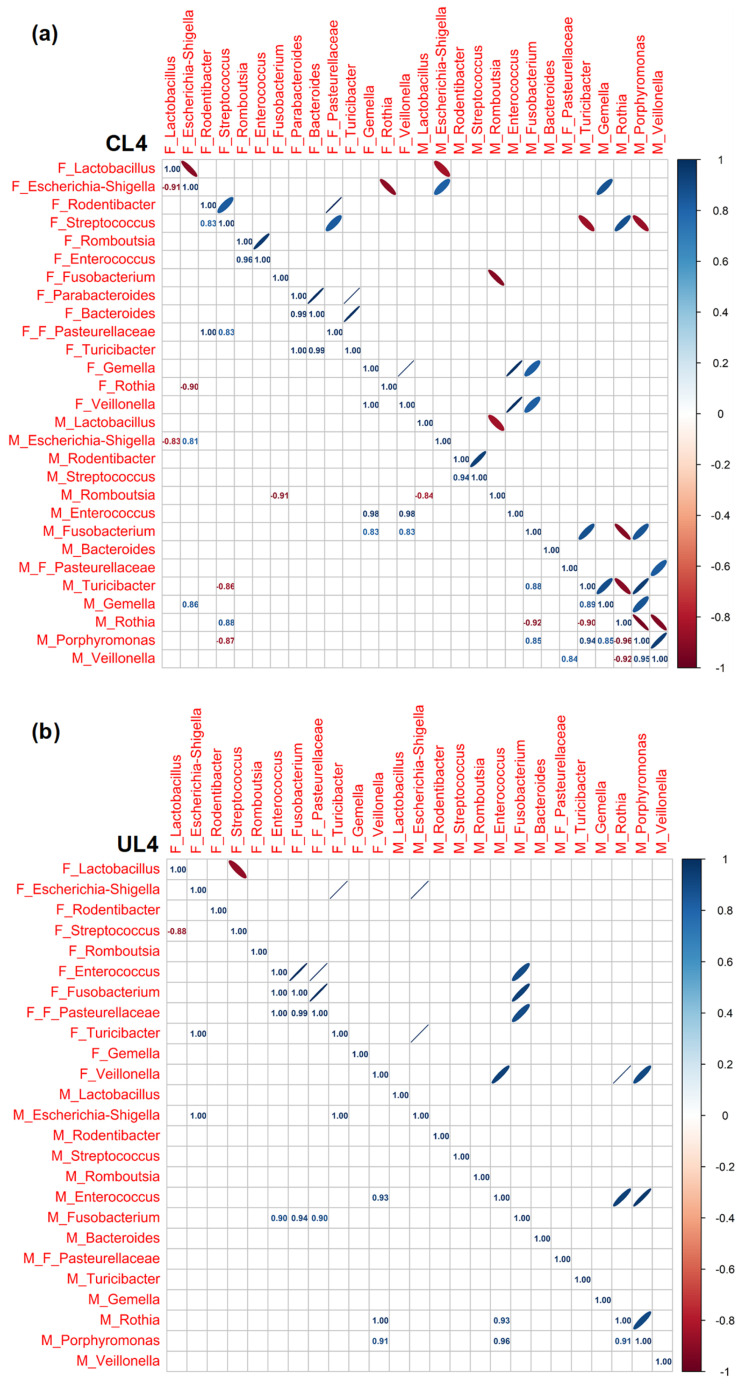
Pearson’s rank correlation matrix of the 14 most abundant bacterial genera in milk and the 15 most abundant bacterial genera in feces of C (**a**) and U (**b**) rats at 4 days of lactation (L4). Red represents a negative correlation and blue represents a positive correlation. C: control; U: undernourished; the F preceding the genera indicates feces origin (F: feces). The M preceding the genera indicates milk origin (M: milk). The F preceding F_Pasteurelleceae indicates the family taxonomic level. N = 5–6.

**Figure 6:**
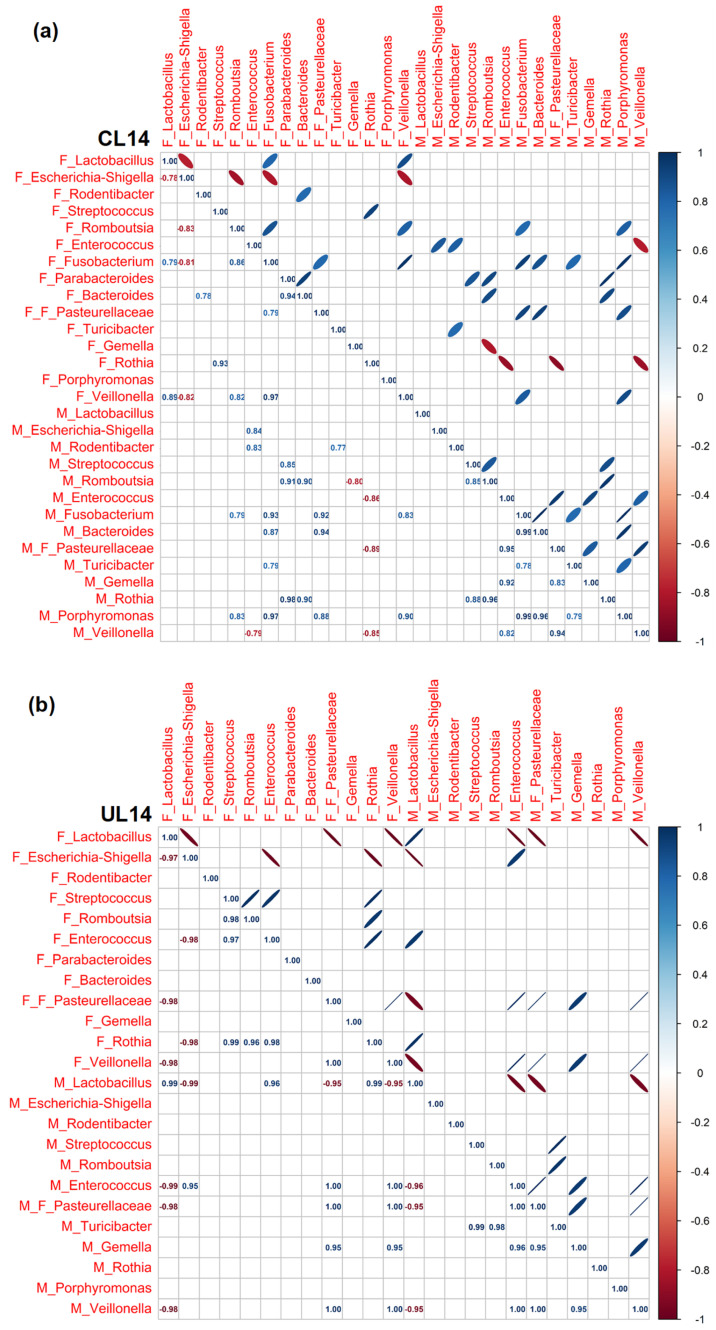
Pearson’s rank correlation matrix of the 14 most abundant bacterial genera in milk and the 15 most abundant bacterial genera in the feces of C (**a**) and U (**b**) rats at 14 days of lactation (L14). Red represents a negative correlation and blue represents a positive correlation. C: control; U: undernourished; the F preceding the genera indicates feces origin (F: feces). The M preceding the genera indicates milk origin (M: milk). The F preceding F_Pasteurelleceae indicates the family taxonomic level. N = 5–6.

**Figure 7:**
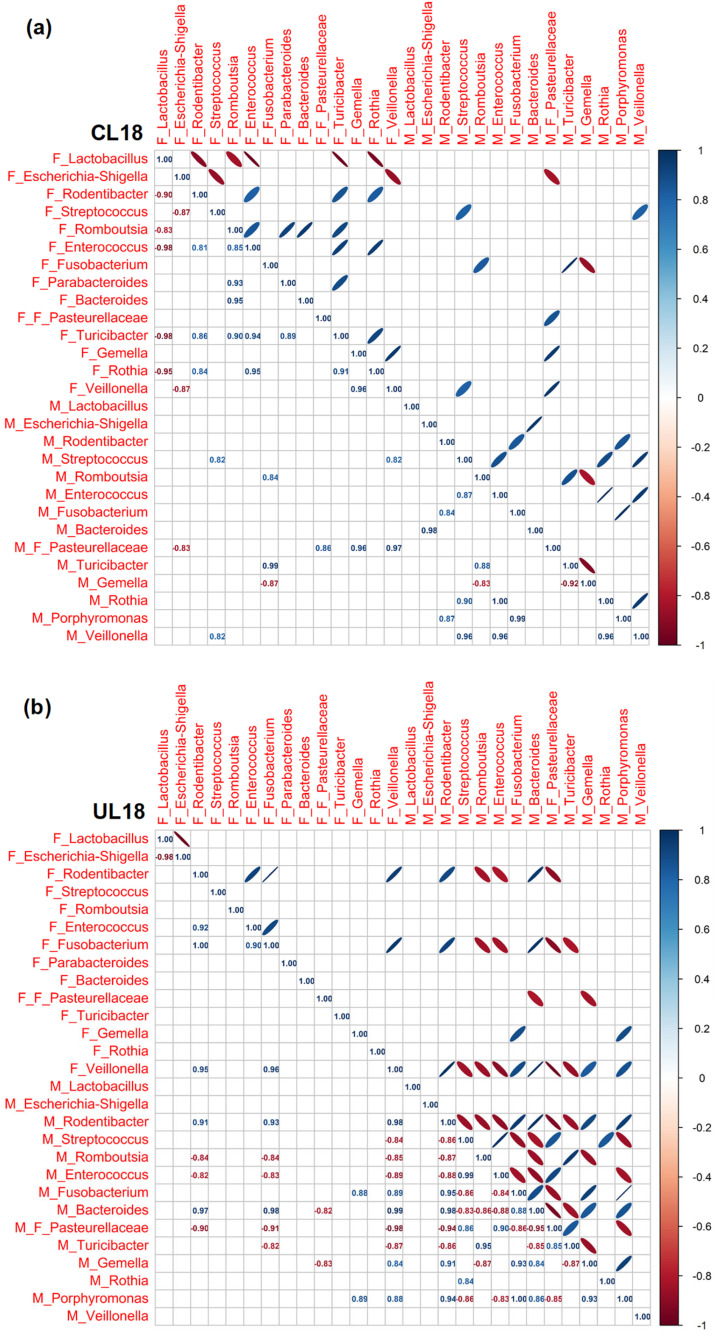
Pearson’s rank correlation matrix of the 14 most abundant bacterial genera in milk and the 15 most abundant bacterial genera in feces of C (**a**) and U (**b**) rats at 18 days of lactation (L18). Red represents a negative correlation and blue represents a positive correlation. C: control; U: undernourished; the F preceding the genera indicates feces origin (F: feces). The M preceding the genera indicates milk origin (M: milk). The F preceding F_Pasteurelleceae indicates the family taxonomic level. N = 5–6.

In the current analysis, we observed correlations between the predominant bacterial genera in both milk and fecal samples over the studied postnatal days. Our data highlighted an evolving landscape of correlations, with changing patterns as the days progressed (Figure 5, Figure 6 and Figure 7).

In general, we found (i) a progressive increase in the number of correlations throughout the lactation period, regardless of the type of diet; (ii) that nutritional restriction seemed to establish stronger associations between genera than those observed in well-nourished animals; (iii) that the correlations were particularly numerous at the end of lactation in both groups of animals although in the C group associations were more frequent among bacteria exclusively from feces, while dietary restriction led to a greater number of associations among milk bacteria. Despite this dynamism, a recurring trend emerged: the presence of *Escherichia-Shigella* in milk samples often mirrored its presence in fecal samples. Furthermore, the analysis brought to light a general trend where *Escherichia-Shigella* and *Lactobacillus* exhibited a negative correlation across various time points, at L4 for C samples (r = −0.91) and at L14 and L18 for U samples (r = −0.98 at both ages). This suggests a pattern where a high prevalence of one genus might be associated with a decreased prevalence of the other. While this recurring trend was observed, it warrants further investigation to explore the potential implications and underlying mechanisms driving these observations. Regarding the positive associations between fecal anaerobic bacteria, an indicator of intestinal maturity, these were more frequent in C animals at the different times studied than they were in U pups (*Parabacteroides*, *Bacteroides*, *Turicibacter*, *Gemella*, *Veillonella*, *Escherichia-Shigella*, *Fusobacterium*, *Lactobacillus*, *Rombustia* and *Rodentibacter*), which again evidenced the influence of diet in gut colonization.

### 3.4. Nutritional Restriction during Lactation Disrupts the Colonic Barrier Integrity of the Offspring

Given the shift in the bacterial profile of malnourished pups, their intestine structural and morphometric characteristics were analyzed. As shown in Table 4, the body (from nose to annus) and total intestinal length (from pylorus to cecum) increased progressively from 4 to 18 days of lactation in both experimental groups C and U; however, the growth experienced by U offspring rats was less pronounced and the measurements were significantly lower than those in C animals. The length of the small intestine in pups from restricted mothers was always lower than the C values but it only had statistical significance at L4 and L18. Regarding the lengths of the large intestine, including colon and cecum, the highest differences with C values were observed at L4 whereas no variations were detected at L14 and L18 for the colon. Surprisingly, the U cecum was found to be longer than the cecum of C rats at L14; however, its length decreased again towards the end of lactation (L18), removing the statistical significance.

Moreover, in order to evaluate if body proportions were maintained despite nutritional restriction, we adjusted the length of the small intestine and colon based on the total body length. The measurements of the small intestine in the U group did not differ from those of the C rats. However, nutritional restriction resulted in significant changes in the fractional length of the colon, which was higher at L14 and L18 compared to that in the age-matched C group. This suggests that there is some preservation of colon growth during this stage of development (Table 4).

**Figure 8:**
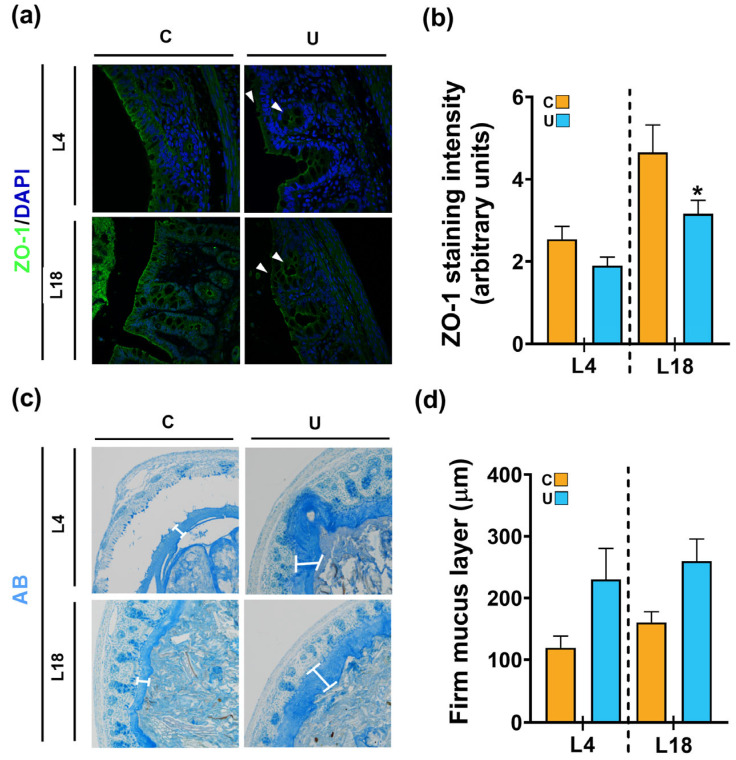
Characteristics of the colonic barrier integrity and firm mucus layer in the offspring rats at 4 and 18 days of lactation. (**a**) Immunofluorescence of ZO-1 (green) in colon sections. Nucleic acids were stained with DAPI (blue) and white arrows indicate areas where the ZO-1 stain was disrupted at the epithelial cell membrane (magnification: 63×). (**b**) Quantification of the ZO-1 fluorescence intensity expressed in arbitrary units. (**c**) Alcian Blue (AB)-stained colonic sections. The firm mucus layer was marked with white brackets (magnification: 20×). (**d**) Quantification of the firm mucus thickness. Data represent mean ± SEM (*n* = 3–4; 4 sections per animal). Student’s *t* analysis was used to evaluate differences between groups in terms of the type of diet. * *p* < 0.05 compared to C group within each age. C: control; U: undernourished; L: lactation.

As the intestinal barrier is immature at birth and undergoes important structural and functional changes induced by the abrupt transition in nutrient supply from maternal umbilical cord blood to enteral milk intake, intestinal permeability and mucus layer thickness were analyzed. These studies focused on the colonic tissue because this is the main microbiological reservoir of the organism. To assess the first parameter, we measured ZO-1 levels, which is a tight junction protein located in the apical zone of colonic epithelial cells that regulates paracellular permeability and contributes to the preservation of intestinal barrier integrity [31]. Immunofluorescence analysis performed at early (L4) and late lactation (L18) showed an increase in signal intensity for ZO-1 in C animals, forming a network of increasingly consolidated integrity in epithelial cells (Figure 8a). However, ZO-1 levels were significantly reduced in U rats at L18 compared to those in the C group (Figure 8b), suggesting a loss of intestinal barrier integrity at this age.

**Figure 9:**
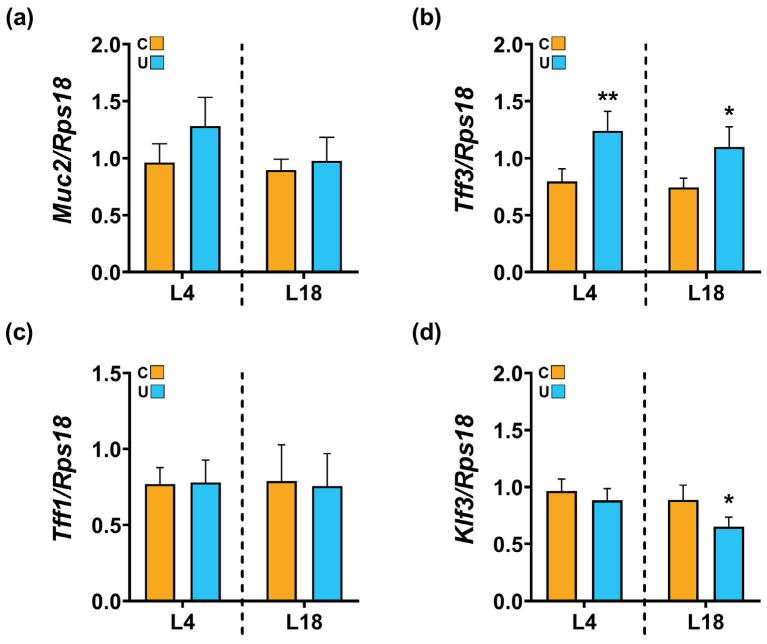
Changes induced by maternal nutrition in the expression profile of goblet cell markers from the offspring colon at 4 and 18 days of lactation. Quantitative RT-qPCR analysis of *Muc2* (**a**), *Tff3* (**b**), *Tff1* (**c**) and *Klf3* (**d**) normalized with the 18S ribosomal gene. Data represent mean ± SEM (*n* = 5–6). Student’s t-analysis was used to evaluate differences between the two groups in term sof the type of diet. * *p* < 0.05; ** *p* < 0.001 compared to group C within each age. C: control; U; Undernourished; L: lactation.

The colonic lumen contains a gel-like structure called mucus that acts as a first line of defense against microorganisms invading the lamina propria. It is produced by specialized secreting cells called goblet cells, which form part of the intestinal epithelium [32]. Because microbial changes also play an important role in mucus production, we then quantified the inner mucus thickness with AB staining (Figure 8c). Morphometric measurements of the mucus layer showed a trend of enhanced thickness under food-restricted conditions at both L4 (1.9-fold increase vs. C) and L18 (1.6-fold increase vs. C); however, this did not reach statistical significance (*p* = 0.068, for both ages) (Figure 8d). To further characterize the effect of maternal nutrition on gut barrier properties, we next analyzed the gene expression of key markers of specialized mucus-secreting goblet cells: *Muc2*, *Tff3*, *Tff1* and *Klf3* (Figure 9). Nutritional restriction did not induce any significant change in the expression of *Muc2*, the main goblet cell marker, relative to C group levels at any age considered (Figure 9a). On the contrary, the expression of *Tff3*, a factor co-secreted with MUC2 and involved in cell migration, proliferation and the repair of the mucosal epithelium, was significantly higher at 4 (1.56-fold increase) and 18 days of life (1.48-fold increase) compared to the C values (Figure 9b). Although the mucosal protective function of TFF1 is similar to that of TFF3, its expression was not altered in U offspring rats relative to that of C animals during lactation (Figure 9c). Finally, *Klf3* expression, another factor involved in the regeneration of epithelial cells, was significantly downregulated at late lactation (L18) under nutrient-restricted conditions (Figure 9d).

## 4. Discussion

Lactation is considered a period of major relevance in metabolic programming because milk bacteria are among the first microbes to enter the neonatal gastrointestinal tract and, consequently, they act as drivers in the acquisition and development of a healthy microbiota [18,33]. Mother-to-infant bacterial transfer through maternal milk has been repeatedly described at the species and/or the strain level, via both culture-dependent and culture-independent techniques [18,34]. Worth noting is that approximately a quarter of the bacteria detected in infant feces during early life seems to derive from milk [35] and the bacterial genera accounting for most of the abundance (>70%) in infant feces are shared with human milk [36]. In the same direction, it has been recently described that maternal milk is closely associated with offspring microbiome diversity [37]. Therefore, as diet has been shown to be a major determinant of the type and abundance of microbiota in the gastrointestinal tract, in the present study we have investigated how maternal diet during pregnancy and lactation affect this microbial communication between mother and offspring. Our data show that the disorder of milk microbiota induced via maternal food restriction seems to change the profile of offspring intestinal microbiome and then promote an immature intestinal microbiota and gut barrier function.

Even though no dietary effect was observed for alpha diversity, neither in milk nor in fecal samples, beta diversity was significant for age and diet, in both types of samples, with the bacteria found in U samples at L4 displaying the highest distance to all other samples. It is worth noting that bacteria identified in fecal samples from UL14 were more similar to CL4 than to those collected from animals of the same age but consuming a different diet. These results reinforce the idea that maternal diet influence milk microbial composition and this effect is mirrored in offspring feces, promoting an immature gut microbiome. Likewise, severely undernourished children from Bangladesh [38] showed marked gut microbiota immaturity that could not be reversed with dietary interventions, which is in agreement with our previous studies performed with a catch-up growth rat model [16] or in humanized mouse models of malnutrition [39].

The bacteria found in milk and offspring feces during lactation were mainly represented by the phyla Firmicutes, Proteobacteria, Bacteroidota and Actinobacteria, the latter taxa being less abundant than the former two. The most abundant ASVs found in the milk were identified as *Lactobacillus*, which accounted for most of the reads within the phylum Firmicutes. The contribution of *Lactobacillus* spp. to milk is highly conserved across species and known to produce bacteriocins, which are able to inhibit the growth of pathogens [40]. Moreover, as a facultative anaerobe, *Lactobacillus* is considered a pioneer bacterial specie that during the first days of lactation depletes oxygen from gut and thereby facilitates the subsequent colonization of the gut environment via obligate anaerobes [41]. Warren et al. [27] reported that variations in the protein content of maternal diet influenced the relative abundance of the genus *Lactobacillus* not only from maternal milk but also from offspring feces. In agreement, we observed *Lactobacillus* spp. to show greater relative abundance at the beginning of lactation in the feces from pups of mothers subjected to food restriction, despite no parallel effect of diet being observed in milk. Interestingly, it has been suggested that higher colonization of the gut with *Lactobacillus* spp. in early life predicts an increased risk of being overweight and metabolic alterations in the future [42].

Given that *Lactobacillus* spp. are normally found in the vaginal niche, it is possible that the high levels of *Lactobacillus* taxa in the offspring fecal samples from U mothers originated in part, during delivery, from changes in the vaginal microbiota composition of mothers. The composition of the vaginal microbiota is under the control of hormonal patterns [43] with higher abundance in *Lactobacillus* species with increasing gestational age. This event seems to be part of a strategy to ensure lactic acid production, reduce pH in the vaginal cavity and protect the fetus from infections. However, the influence of environmental factors, such as stress or diet, on vaginal community stability is poorly known. Although chronic stress induced by severe food restriction, like in patients with anorexia nervosa, has been associated with bacterial vaginosis, menstrual cycle irregularity and amenorrhea [44], the interaction between diet, menstrual dysfunction and vaginal community stability remain unexplored. Thus, it is possible that maternal diet during gestation may alter the vaginal microbiota transmitted to offspring and subsequently influence their gut colonization and maturation.

In addition to *Lactobacillus*, *Escherichia-Shigella*, included in the phylum Proteobacteria, is also considered a pioneer bacterium with high abundance throughout lactation in the feces of well-nourished pups. However, its proliferation appears to be conditioned by nutritional status given its almost absolute absence at the beginning of lactation in the milk of U mothers as well as in their offspring feces, but the subsequent sharp increase in feces. This event might affect the establishment of a suitable intestinal microenvironment to ensure the colonization of strict anaerobic bacteria and consequently, delay gut microbiota maturation. Although *Escherichia-Shigella* is a non-pathogenic genus, under specific circumstances, certain serotypes can trigger symptoms such as diarrhea, with increased intestinal permeability and disrupted epithelial barrier or even extra-intestinal inflammatory processes [45,46,47,48]. The low number of samples analyzed in this study implies that the microbial shifts detected for this genus did not reach a statistically significant threshold but might, in fact, be relevant for homeostasis and health, and should be carefully evaluated in future studies involving a larger number of animals.

Within the phylum Proteobacteria, our metataxonomic analysis also successfully detected sequences belonging to the family *Pasteurellaceae*. Most of them were assigned to the genus *Rodentibacter*, while a minor fraction was assigned to other genera, such as *Haemophilus* and *Muribacter*. However, it is important to note that our sequencing approach faced some limitations in distinguishing between sequences belonging to different genera within this family. Consequently, although the elevated abundance of *Pasteurellaceae* in milk samples from U animals at L4 might primarily be attributable to *Rodentibacter*, the potential contribution of other genera within this family should not be discarded.

It is noteworthy that in the present study, mothers’ milk was not collected from the mammary gland as is carried out in other similar approaches [27], but from the pup’s stomach, which is indeed the same route as that for suckling offspring. Undoubtedly, the offspring microbiota, particularly that from the oral cavity, will modify the bacteria expressed in milk [19,49], but will also contribute to the establishment of the first gut colonizers. In line with this concept, the presence of the genus *Porphyromonas* in the milk of nutritionally restricted mothers drew attention in our studies. *Porphyromonas* is an anaerobic bacterium found in the oral cavity and is associated with the development of periodontal disease. Its presence in the oral cavity and consequently, its ingestion during food intake causes intestinal dysbiosis by creating an environment favorable to the colonization of pro-inflammatory microorganisms [50] and eventually leading to ulcerative colitis [51] or colorectal cancer [52]. Likewise, metabolic studies performed with streptozotocin-treated mice (to generate a diabetic situation) and subsequent *Porphyromonas* administration showed a worsening of the glycemic control together with local and systemic inflammatory events [53]. Thus, the presence of *Porphyromonas* in milk might be considered a biomarker of future gut dysbiosis, inflammatory processes and long-term metabolic alterations. Accordingly, we have previously described the aforementioned etiopathological triangle in the same animal model of food restriction used herein [16]. However, the very early triggering events of this phenotype and its relationship with breastfeeding and maternal diet needed further investigations.

On the other hand, it is known that intestinal permeability at birth is high in newborns and then gradually decreases to ensure full functionality and protection from pathogens [54]. Moreover, evidence suggest that certain bacteria and bioactive compounds in breast milk play a key role in regulating mucosal integrity and intestinal epithelial permeability. Human studies have shown that full-term breastfed infants have lower intestinal permeability than do formula-fed infants [55,56]. These observations are consistent with animal studies showing that germ-free mice have many morphological intestinal defects compared to their normally colonized counterparts, suggesting that the development of barrier function is dependent on the presence of microorganisms [57]. However, the specific mechanisms by which variations in the gut microbiota shape the early plasticity of intestinal permeability and how these factors contribute to health status in later life remain poorly understood. The apical epithelial network of tight junction proteins is the principal determinant of paracellular permeability and immune system homeostasis [31]. In the present study, we observed that maternal food restriction increased colonic permeability in the lactating offspring, as evidenced by a reduction in ZO-1 protein levels, which was not fully ameliorated when undernutrition was corrected postweaning [16]. In agreement, severe protein deficiency in animal models led to structural and functional changes of the intestine, including malabsorption and the development of a leaky gut with increased permeability [17,58]. Intestinal biopsies from severely undernourished children with enteropathy also showed a reduced expression of the tight junctions claudin-4 and E-cadherin [59], although the molecular mechanism relating undernutrition to changes in paracellular proteins remains unclear. In line with this, the disruption of epithelial tight junction proteins is consistent with the translocation of bacterial components to the circulation and development of endotoxemia, as we [16] and others [60] have previously described. These changes in tight junction protein levels paralleled changes in the expression of epithelial cell components such as the trefoil factor family of peptides (TFF), which are normally co-secreted with mucins. In particular, TFF1 and TFF3 actively participate with immune system molecules in protecting the intestinal mucosa from microorganisms [61]. In our animal model, we did not observe differences in *Tff1* expression associated with diet type. However, at the beginning and end of lactation, the expression of *Tff3* was increased in offspring subjected to dietary restriction. In this regard, recent studies in animals and pediatric patients with inflammatory bowel disease have shown elevated serum levels of TFF3, an event associated with increased intestinal permeability and inflammation that is not seen in healthy subjects or patients in remission [62]. Therefore, considering the lack of differences in mucin (*Muc2*) expression between C and U lactating pups, but the increase in *Tff3* in the latter, we hypothesize that the inflammatory response is activated in the colon of this population. In line with this, Fança-Berthon et al. [17] previously described that the offspring of mothers fed a low-protein diet showed disturbed expression of *Tff3* in the colon during lactation. Likewise, the KLF (Krüppel-like factor) family of transcription factors is also involved in the control of cell proliferation and differentiation in both healthy and pathological situations, as well as in inflammatory responses [63]. Moreover, studies in humans with colorectal cancer have described an unfavorable prognosis at low expression levels of KLF3 [64]. Therefore, the reduced expression of this factor in the colon of UL18 rats compared to that in CL18 animals could be considered a marker of incipient inflammation. Taken together, these results suggest that increased permeability may provide transitional benefits for U pups, such as improved nutrient uptake and the development of systemic tolerance, which might favor their survival. However, it may also imply some disadvantages, including the increased translocation of microbes and foreign particles leading to the development of infection, inflammation and metabolic disease.

Finally, we used Pearson’s correlations to support vertical mother-to-child transmission and the potential influence of nutritional status in this process. It should be noted that vertical transmission can be direct, when the presence of a particular bacterium in the milk determines the presence of the same genus in the feces, or indirect, when the presence of a particular genus in the milk determines the presence or absence of other different genera in the feces. Thus, at early lactation, the presence of *Escherichia-Shigella* in the milk from well-fed animals positively correlated with the abundance of these taxa in the offspring’s feces, but with a lower intestinal growth of *Lactobacillus* spp. This negative correlation between milk-derived *Escherichia-Shigella* and the fecal content of *Lactobacillus* was not observed in U animals, probably due to the discriminant abundance of *Escherichia-Shigella* in the milk of food-restricted mothers at L4 which might have allowed the initial colonization of the gut to be dominated by *Lactobacillus* spp. However, the later reduction of this taxon in the milk from food-restricted animals, strongly correlated with lower presence of *Lactobacillus* in feces, but the higher relative abundance of *Escherichia-Shigella* supported the negative correlation between the relative abundance of these two genera in feces as previously described during the progression from ulcerative colitis to colorectal cancer [65]. Interestingly, Pearson’s correlations also evidenced that the number of positive associations detected during lactation between fecal anaerobic bacteria, including *Fusobacterium*, *Rodentibacter*, *Bacteroides*, *Rombustia* or *Veillonella* was significantly more abundant in control animals than in those exposed to maternal food restriction, suggesting that the formation of an anaerobic environment, characteristic of an adult gut microbial profile, was delayed in the offspring as a result of maternal diet. Thus, our findings indicate that gut microbial composition is not only determined by the particular characteristics of each microbial community, but that environmental changes also shape the final microbiota.

Several advantages and limitations of the present study should be noted. The main strength is that by collecting sequential samples from each litter, we could examine longitudinal changes in milk microbiota composition over time and correlate them with variations in the fecal microbiome. In addition, the use of a rat model offers the advantage of being able to carefully control the maternal diet in the absence of other external influences to study the intestinal outcomes of the offspring. However, this in itself is a limitation when we consider that rats are polytocous and altricial mammals, meaning that rats give birth to large litters born at an immature stage after relatively short pregnancies, whereas women generally bear a single fetus at an advanced stage of metabolic development. However, comparative physiology between species provides an opportunity to understand the molecular, cellular and biochemical mechanisms involved in the early origin of metabolic programming. On the other hand, diet and age were not able to explain all the changes observed in milk and fecal bacterial composition, suggesting that other factors may have contributed to the large inter-individual variations observed in the microbiota profiles. In this regard, sex differences in the gut microbiota have been reported in adult individuals and are usually attributed to hormonal differences between males and females. However, there is no consensus on this relationship in early life colonization or in relation to breastfeeding [42,49]. Thus, the absence of gender-stratified analysis in the present study could possibly explain why our data showed some inconclusive results on the association of the diet-induced shift in the milk microbiota with variations in the fecal composition of the offspring. Finally, as 16S rRNA gene sequencing has limited ability to resolve taxa beyond the genus level, further metagenomic and culturing analysis will be required to confirm and validate the results of this study as well as to quantify bacterial load and the viability of the bacteria identified in our samples.

## 5. Conclusions

**Figure 10:**
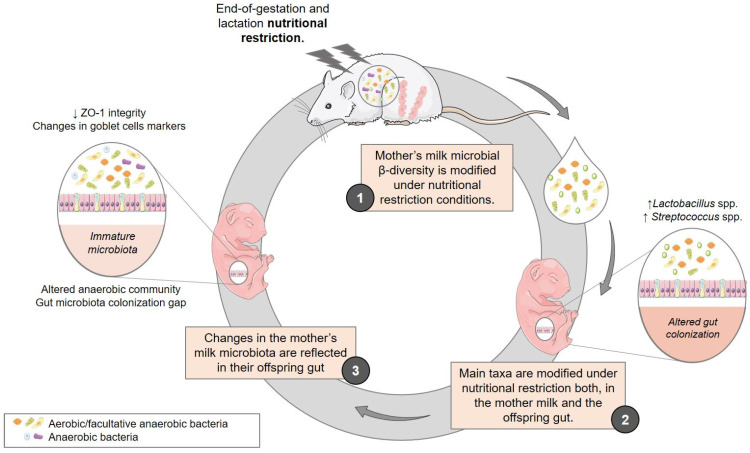
Maternal nutrition determining milk microbiota and influencing offspring gut colonization. Nutritional restriction during the early stages of development alters the microbial composition of maternal milk, a phenomenon reflected in the intestinal microbiota of the offspring. As a result, it alters the abundance of major taxa in milk and feces, delaying the maturing of offspring gut microbiota. Additionally, these changes in microbial composition are associated with changes in gut permeability and in the goblet cells markers, which are associated with chronic regeneration of cells and inflammatory processes.

In conclusion (Figure 10), maternal malnutrition is able to modify the microbial composition of breastmilk, a phenomenon that is reflected in the intestinal microbiota of the offspring. This fact supports the idea of a vertical transmission of bacteria from mother to child. One of the effects of this nutritional condition is the delay in the maturation of the intestinal microbiota, as evidenced by the gap that exists both, in the evolution of the bacterial diversity and the appearance of the main colonizing genera during lactation. These microbial changes were accompanied by alterations in offspring gut permeability, particularly at the end of lactation, as well as in the goblet cell markers *Tff3* and *Klf3*, associated with cell renewal and chronic inflammatory processes. It is worth mentioning that our previous studies [16] have already demonstrated that this microbiota immaturity is maintained beyond weaning and even until adulthood, which points out the potential contribution of altered gut colonization to the associated morbidities and the sequelae of malnutrition in early life, including an increased risk of obesity and glucose intolerance. Therefore, our data highlight the importance of breastfeeding as a critical window for therapeutic intervention via the modulation of the infant microbiota as part of preventive medicine.

## Figures and Tables

**Table 1:** List of primers (Sigma-Aldrich).

Gene	Symbol	Sequence (5′–3′)
MUC2	*Muc2*	AAGCCAGATCCCGAAACCAT
		ATGGCCCCATTCACAACTGCC
TFF1	*Tff1*	CAAGGTGACCTGTGTCCTC
		CTTGCTGGTTCTCAATGACC
TFF3	*Tff3*	GACTCCAGCATCCCAAATGT
		GCAGATCAGGGGTGAGTGTT
KLF3	*Klf3*	TCATGTACACCAGCCACCTG
		TAGTCAGTCCTCTGTGGTTC
18S ARNr	*Rps18*	GTAACCCGTTGAACCCCATT
		CCATCCAATCGGTAGTAGCG

**Table 2:** Characteristics of the litters at birth.

	Control	Undernourished
Newborns weight (g)	6.16 ± 0.14	5.69 ± 0.09 **
Litter size	11.5 ± 1.30	9.86 ± 0.72
Number of male newborns	6.25 ± 11.11	5.71 ± 0.65
Number of female newborns	5.25 ± 0.63	4.00 ± 0.67
Ratio males/females	1.23 ± 0.24	1.88 ± 0.25

Data are means ± SEM (6–8 animals). Student’s *t* analysis was used to evaluate differences between the two groups. ** *p* < 0.01 compared to C group.

**Table 3:** Serum biochemical profile of the offspring during lactation.

	Control			Undernourished		
	L4	L14	L18	L4	L14	L18
Body weight (g)	11.36 ± 0.23	27.94 ± 0.4	34.65 ± 1.02	8.35 ± 0.15 ***	16.5 ± 0.41 ***	16.62 ± 0.36 ***
Glycemia (mg/dL)	119.1 ± 1.43	107.8 ± 1.03	146.19 ± 1.19	85.9 ± 4.02 ***	85.3 ± 5.29 **	93.15 ± 4.94 ***
Insulinemia (ng/mL)	0.67 ± 0.06	0.70 ± 0.08	0.78 ± 0.17	0.29 ± 0.04 ***	0.25 ± 0.06 **	0.36 ± 0.09 *
Glucagonemia (pg/mL)	252.3 ± 10.6	298 ± 11.2	129.1 ± 6.9	103.9 ± 4.4 ***	97 ± 1.3 ***	83.2 ± 4.3 ***
Cholesterol (mg/dL)	154.66 ± 4.11	127.48 ± 5.81	119.95 ± 4.5	144.38 ± 3.28	119.62 ± 4.51	116.86 ± 5.2
Triglycerides (mg/dL)	157.08 ± 5.26	79.94 ± 4.75	63.41 ± 5.42	160.17 ± 8.57	74.02 ± 3.07	68.42 ± 3.51

Data are mean ± SEM (*n* = 6–8 animals). Biochemical and hormonal parameters of control (C) and undernourished (U) rats at 4, 14 and 18 days of lactation (L). Student’s *t* analysis was used to evaluate differences between groups in terms of nutritional type. * *p* < 0.05; ** *p* < 0.01; *** *p* < 0.001 compared to C group at the same age.

**Table 4:** Intestinal morphometric characteristics of the offspring during lactation.

	Control			Undernourished		
	L4	L14	L18	L4	L14	L18
Body length (cm)	6.23 ± 0.07	8.89 ± 0.09	9.36 ± 0.114	6.28 ± 0.17	7.4 ± 0.09 ***	8.01 ± 0.13 ***
Total intestine length (cm)	33.47 ± 0.37	48.0 ± 0.90	48.43 ± 1.53	29.54 ± 0.67	41.0 ± 2.30	39.41 ± 0.72 ***
Small intestine length (cm) Colon length (cm) Cecum length (cm)	29.15 ± 0.38	40.6 ± 0.81	41.47 ± 1.34	25.27 ± 0.64 **	35.0 ± 2.20	33.14 ± 0.70 ***
	3.64 ± 0.13	5.83 ± 0.14	5.90 ± 0.24	3.16 ± 0.12 *	5.17 ± 0.19	5.52 ± 0.14
	0.68 ± 0.04	1.35 ± 0.06	1.38 ± 0.05	0.65 ± 0.03 **	1.43 ± 0.08	1.26 ± 0.05
Small Intes. Length/Body Length	4.68 ± 0.14	4.57 ± 0.12	0.48 ± 0.19	4.64 ± 0.10	5.23 ± 0.32	4.49 ± 0.20
Colon Length/Body Length	0.56 ± 0.03	0.67 ± 0.01	0.63 ± 0.03	0.57 ± 0.02	0.76 ± 0.03 *	0.75 ± 0.03 **

Quantification of intestinal parameters of the offspring at 4, 14 and 18 days of lactation (L4, L14 and L18). Student’s t-analysis was used to evaluate differences between groups in terms of nutrition type. Data represent mean ± SEM (*n* = 6–8). * *p* < 0.05; ** *p* < 0.01; *** *p* < 0.001 compared to C group.

## Data Availability

Data are available upon request to the authors.

## Supplementary Materials

The following supporting information can be downloaded at https://www.mdpi.com/article/10.3390/nu15204322/s1. Table S1: Relative abundance of the most abundant genera in the milk of control (C) and undernourished (U) rats at 4, 14 and 18 days of lactation.; Table S2: Relative abundance of the most abundant genera in the feces of control (C) and undernourished (U) offspring rats at 4, 14 and 18 days of lactation.

## References

[1] Pena-León V., Folgueira C., Barja-Fernández S., Pérez-Lois R., Da Silva Lima N., Martin M., Heras V., Martínez-Martínez S., Valero P., Iglesias C. (2022). Prolonged breastfeeding protects from obesity by hypothalamic action of hepatic FGF21. Nat. Metab..

[2] Picó C., Reis F., Egas C., Mathias P., Matafome P. (2021). Lactation as a programming window for metabolic syndrome. Eur. J. Clin. Investig..

[3] Hsu C.N., Hou C.Y., Hsu W.H., Tain Y.L. (2021). Early-Life Origins of Metabolic Syndrome: Mechanisms and Preventive Aspects. Int. J. Mol. Sci..

[4] De Toro-Martín J., Fernández-Millán E., Lizárraga-Mollinedo E., López-Oliva E., Serradas P., Escrivá F., Álvarez C. (2014). Predominant role of GIP in the development of a metabolic syndrome-like phenotype in female Wistar rats submitted to forced catch-up growth. Endocrinology.

[5] Tsuduki T., Kitano Y., Honma T., Kijima R., Ikeda I. (2013). High dietary fat intake during lactation promotes development of diet-induced obesity in male offspring of mice. J. Nutr. Sci. Vitaminol..

[6] Barker D.J. (2007). The origins of the developmental origins theory. J. Intern. Med..

[7] Grigor M.R., Allan J.E., Carrington J.M., Carne A., Geursen A., Young D., Thompson M.P., Haynes E.B., Coleman R.A. (1987). Effect of Dietary Protein and Food Restriction on Milk Production and Composition, Maternal Tissues and Enzymes in Lactating Rats. J. Nutr..

[8] Bautista C.J., Bautista R.J., Montaño S., Reyes-Castro L.A., Rodriguez-Peña O.N., Ibáñez C.A., Nathanielsz P.W., Zambrano E. (2019). Effects of maternal protein restriction during pregnancy and lactation on milk composition and offspring development. Br. J. Nutr..

[9] Wattez J.S., Delmont A., Bouvet M., Beseme O., Goers S., Delahaye F., Laborie C., Lesage J., Foligné B., Breton C. (2015). Maternal perinatal undernutrition modifies lactose and serotranferrin in milk: Relevance to the programming of metabolic diseases?. Am. J. Physiol. Endocrinol. Metab..

[10] Fiorotto M.L., Burrin D.G., Perez M., Reeds P.J. (1991). Intake and use of milk nutrients by rat pups suckled in small, medium, or large litters. Am. J. Physiol..

[11] Palou M., Torrens J.M., Castillo P., Sánchez J., Palou A., Picó C. (2020). Metabolomic approach in milk from calorie-restricted rats during lactation: A potential link to the programming of a healthy phenotype in offspring. Eur. J. Nutr..

[12] Lizárraga-Mollinedo E., Fernández-Millán E., de Toro Martín J., Martínez-Honduvilla C., Escrivá F., Álvarez C. (2012). Early undernutrition induces glucagon resistance and insulin hypersensitivity in the liver of suckling rats. Am. J. Physiol. Metab..

[13] de Toro-Martín J., Fernández-Marcelo T., González-Rodríguez Á., Escrivá F., Valverde Á.M., Álvarez C., Fernández-Millán E. (2020). Defective liver glycogen autophagy related to hyperinsulinemia in intrauterine growth-restricted newborn Wistar rats. Sci. Rep..

[14] Lizárraga-Mollinedo E., Fernández-Millán E., García-San Frutos M., de Toro-Martín J., Fernández-Agulló T., Ros M., Álvarez C., Escrivá F. (2015). Early and long-term undernutrition in female rats exacerbates the metabolic risk associated with nutritional rehabilitation. J. Biol. Chem..

[15] López M., Seoane L.M., Tovar S., García M.C., Nogueiras R., Diéguez C., Señarís R.M. (2005). A possible role of neuropeptide Y, agouti-related protein and leptin receptor isoforms in hypothalamic programming by perinatal feeding in the rat. Diabetologia.

[16] Martínez-Oca P., Robles-Vera I., Sánchez-Roncero A., Escrivá F., Pérez-Vizcaíno F., Duarte J., Álvarez C., Fernández-Millán E. (2020). Gut DYSBIOSIS and altered barrier function precedes the appearance of metabolic syndrome in a rat model of nutrient-induced catch-up growth. J. Nutr. Biochem..

[17] Fança-Berthon P., Michel C., Pagniez A., Rival M., Van Seuningen I., Darmaun D., Hoebler C. (2009). Intrauterine growth restriction alters postnatal colonic barrier maturation in rats. Pediatr. Res..

[18] Dogra S.K., Kwong Chung C., Wang D., Sakwinska O., Colombo Mottaz S., Sprenger N. (2021). Nurturing the Early Life Gut Microbiome and Immune Maturation for Long Term Health. Microorganisms.

[19] Xiao L., Zhao F. (2023). Microbial transmission, colonisation and succession: From pregnancy to infancy. Gut.

[20] Lackey K.A., Williams J.E., Meehan C.L., Zachek J.A., Benda E.D., Price W.J., Foster J.A., Sellen D.W., Kamau-Mbuthia E.W., Kamundia E.W. (2019). What’s normal? microbiomes in human milk and infant feces are related to each other but vary geographically: The INSPIRE study. Front. Nutr..

[21] Togo A., Dufour J.-C., Lagier J.-C., Dubourg G., Raoult D., Million M. (2019). Repertoire of human breast and milk microbiota: A systematic review. Future Microbiol..

[22] Stewart C.J., Ajami N.J., O’Brien J.L., Hutchinson D.S., Smith D.P., Wong M.C., Ross M.C., Lloyd R.E., Doddapaneni H., Metcalf G.A. (2018). Temporal development of the gut microbiome in early childhood from the TEDDY study. Nature.

[23] Cabrera-Rubio R., Mira-Pascual L., Mira A., Collado M.C. (2015). Impact of mode of delivery on the milk microbiota composition of healthy women. J. Dev. Orig. Health Dis..

[24] Cabrera-Rubio R., Collado M.C., Laitinen K., Salminen S., Isolauri E., Mira A. (2012). The human milk microbiome changes over lactation and is shaped by maternal weight and mode of delivery. Am. J. Clin. Nutr..

[25] Collado M.C., Laitinen K., Salminen S., Isolauri E. (2012). Maternal weight and excessive weight gain during pregnancy modify the immunomodulatory potential of breast milk. Pediatr. Res..

[26] Kalliomäki M., Collado M.C., Salminen S., Isolauri E. (2008). Early differences in fecal microbiota composition in children may predict overweight. Am. J. Clin. Nutr..

[27] Warren M.F., Hallowell H.A., Higgins K.V., Liles M.R., Hood W.R. (2019). Maternal Dietary Protein Intake Influences Milk and Offspring Gut Microbial Diversity in a Rat (*Rattus norvegicus*) Model. Nutrients.

[28] Cabinian A., Sinsimer D., Tang M., Zumba O., Mehta H., Toma A., Sant’Angelo D., Laouar Y., Laouar A. (2016). Transfer of Maternal Immune Cells by Breastfeeding: Maternal Cytotoxic T Lymphocytes Present in Breast Milk Localize in the Peyer’s Patches of the Nursed Infant. PLoS ONE.

[29] Rodríguez-Cruz M., Alba C., Aparicio M., Checa M.Á., Fernández L., Rodríguez J.M. (2020). Effect of Sample Collection (Manual Expression vs. Pumping) and Skimming on the Microbial Profile of Human Milk Using Culture Techniques and Metataxonomic Analysis. Microorganisms.

[30] Schindelin J., Arganda-Carreras I., Frise E., Kaynig V., Longair M., Pietzsch T., Preibisch S., Rueden C., Saalfeld S., Schmid B. (2012). Fiji: An open-source platform for biological-image analysis. Nat. Methods.

[31] Odenwald M.A., Turner J.R. (2012). The intestinal epithelial barrier: A therapeutic target?. Nat. Rev. Gastroenterol. Hepatol..

[32] Crosnier C., Stamataki D., Lewis J. (2006). Organizing cell renewal in the intestine: Stem cells, signals and combinatorial control. Nat. Rev. Genet..

[33] Milani C., Duranti S., Bottacini F., Casey E., Turroni F., Mahony J., Belzer C., Delgado Palacio S., Arboleya Montes S., Mancabelli L. (2017). The First Microbial Colonizers of the Human Gut: Composition, Activities, and Health Implications of the Infant Gut Microbiota. Microbiol. Mol. Biol. Rev..

[34] Fernández L., Pannaraj P.S., Rautava S., Rodríguez J.M. (2020). The Microbiota of the Human Mammary Ecosystem. Front. Cell. Infect. Microbiol..

[35] Pannaraj P.S., Li F., Cerini C., Bender J.M., Yang S., Rollie A., Adisetiyo H., Zabih S., Lincez P.J., Bittinger K. (2017). Association between breast milk bacterial communities and establishment and development of the infant gut microbiome. JAMA Pediatr..

[36] Murphy K., Curley D., O’Callaghan T.F., O’Shea C.A., Dempsey E.M., O’Toole P.W., Ross R.P., Ryan C.A., Stanton C. (2017). The composition of human milk and infant fecal microbiota over the first three months of life: A pilot study. Sci. Rep..

[37] Yelverton C.A., Killeen S.L., Feehily C., Moore R.L., Callaghan S.L., Geraghty A.A., Byrne D.F., Walsh C.J., Lawton E.M., Murphy E.F. (2023). Maternal breastfeeding is associated with offspring microbiome diversity; a secondary analysis of the MicrobeMom randomized control trial. Front. Microbiol..

[38] Subramanian S., Huq S., Yatsunenko T., Haque R., Mahfuz M., Alam M.A., Benezra A., Destefano J., Meier M.F., Muegge B.D. (2014). Persistent gut microbiota immaturity in malnourished Bangladeshi children. Nature.

[39] Smith M.I., Yatsunenko T., Manary M.J., Trehan I., Mkakosya R., Cheng J., Kau A.L., Rich S.S., Concannon P., Mychaleckyj J.C. (2013). Gut microbiomes of Malawian twin pairs discordant for kwashiorkor. Science.

[40] Martín R., Olivares M., Marín M., Xaus J., Fernández L., Rodríguez J. (2005). Characterization of a reuterin-producing *Lactobacillus coryniformis* strain isolated from a goat’s milk cheese. Int. J. Food Microbiol..

[41] Martino C., Dilmore A.H., Burcham Z.M., Metcalf J.L., Jeste D., Knight R. (2022). Microbiota succession throughout life from the cradle to the grave. Nat. Rev. Microbiol..

[42] Kozyrskyj A.L., Kalu R., Koleva P.T., Bridgman S.L. (2016). Fetal programming of overweight through the microbiome: Boys are disproportionately affected. J. Dev. Orig. Health Dis..

[43] Jašarević E., Bale T.L. (2019). Prenatal and postnatal contributions of the maternal microbiome on offspring programming. Front. Neuroendocrinol..

[44] Milano W., Ambrosio P., Carizzone F., De Biasio V., Foia M.G., Saetta B., Milano M.F., Capasso A. (2022). Menstrual Disorders Related to Eating Disorders. Endocr. Metab. Immune Disord. Drug Targets.

[45] Ribet D., Cossart P. (2015). How Bacterial Pathogens Colonize Their Hosts and Invade Deeper Tissues. Microbes Infect..

[46] Liang H., Song H., Zhang X., Song G., Wang Y., Ding X., Duan X., Li L., Sun T., Kan Q. (2022). Metformin attenuated sepsis-related liver injury by modulating gut microbiota. Emerg. Microbes Infect..

[47] Li X., He C., Li N., Ding L., Chen H., Wan J., Yang X., Xia L., He W., Xiong H. (2020). The interplay between the gut microbiota and NLRP3 activation affects the severity of acute pancreatitis in mice. Gut Microbes.

[48] Cattaneo A., Cattane N., Galluzzi S., Provasi S., Lopizzo N., Festari C., Ferrari C., Guerra U.P., Paghera B., Muscio C. (2017). Association of brain amyloidosis with pro-inflammatory gut bacterial taxa and peripheral inflammation markers in cognitively impaired elderly. Neurobiol. Aging.

[49] Moossavi M., Sepehri S., Robertson B., Bode L., Goruk S., Field C.J., Lix L.M., de Souza R.J., Becker A.B., Mandhane P.J. (2019). Composition and Variation of the Human Milk Microbiota Are Influenced by Maternal and Early-Life Factors. Cell Host Microbe.

[50] Zenobia C., Hajishengallis G. (2015). Porphyromonas gingivalis virulence factors involved in subversion of leukocytes and microbial dysbiosis. Virulence.

[51] Zhao X., Liu J., Zhang C., Yu N., Lu Z., Zhang S., Li Y., Li Q., Liu J., Liu D. (2021). Porphyromonas gingivalis exacerbates ulcerative colitis via Porphyromonas gingivalis peptidylarginine deiminase. Int. J. Oral Sci..

[52] Koliarakis I., Messaritakis I., Nikolouzakis T.K., Hamilos G., Souglakos J., Tsiaoussis J. (2019). Oral Bacteria and Intestinal Dysbiosis in Colorectal Cancer. Int. J. Mol. Sci..

[53] Ohtsu A., Takeuchi Y., Katagiri S., Suda W., Maekawa S., Shiba T., Komazaki R., Udagawa S., Sasaki N., Hattori M. (2019). Influence of Porphyromonas gingivalis in gut microbiota of streptozotocin-induced diabetic mice. Oral Dis..

[54] Kerr C.A., Grice D.M., Tran C.D., Bauer D.C., Li D., Hendry P., Hannan G.N. (2015). Early life events influence whole-of-life metabolic health via gut microflora and gut permeability. Crit. Rev. Microbiol..

[55] Taylor S.N., Basile L.A., Ebeling M., Wagner C.L. (2009). Intestinal permeability in preterm infants by feeding type: Mother’s milk versus formula. Breastfeed Med..

[56] Catassi C., Bonucci A., Coppa G.V., Carlucci A., Giorgi P.L. (1995). Intestinal permeability changes during the first month—Effect of natural versus artificial feeding. J. Pediatr. Gastroenterol. Nutr..

[57] Edwinson A.L., Yang L., Peters S., Hanning N., Jeraldo P., Jagtap P., Simpson J.B., Yang T.Y., Kumar P., Mehta S. (2022). Gut microbial β-glucuronidases regulate host luminal proteases and are depleted in irritable bowel syndrome. Nat. Microbiol..

[58] Ling C., Versloot C.J., Arvidsson Kvissberg M.E., Hu G., Swain N., Horcas-Nieto J.M., Miraglia E., Thind M.K., Farooqui A., Gerding A. (2023). Rebalancing of mitochondrial homeostasis through an NAD+-SIRT1 pathway preserves intestinal barrier function in severe malnutrition. EBioMedicine.

[59] Amadi B., Besa E., Zyambo K., Kaonga P., Louis-Auguste J., Chandwe K., Tarr P.I., Denno D.M., Nataro J.P., Faubion W. (2017). Impaired barrier function and autoantibody generation in malnutrition enteropathy in Zambia. EBioMedicine.

[60] Patterson G.T., Osorio E.Y., Peniche A., Dann S.M., Cordova E., Preidis G.A., Suh J.H., Ito I., Saldarriaga O.A., Loeffelholz M. (2022). Pathologic Inflammation in Malnutrition Is Driven by Proinflammatory Intestinal Microbiota, Large Intestine Barrier Dysfunction, and Translocation of Bacterial Lipopolysaccharide. Front. Immunol..

[61] Lieleg O., Lieleg C., Bloom J., Buck C.B., Ribbeck K. (2012). Mucin Biopolymers As Broad-Spectrum Antiviral Agents. Biomacromolecules.

[62] Teng X., Yang Y., Liu L., Yang L., Wu J., Sun M., Xu L. (2020). Evaluation of inflammatory bowel disease activity in children using serum trefoil factor peptide. Pediatr. Res..

[63] Bureau C., Hanoun N., Torrisani J., Vinel J.-P., Buscail L., Cordelier P. (2009). Expression and Function of Kruppel Like-Factors (KLF) in Carcinogenesis. Curr. Genom..

[64] Wang X., Jiang Z., Zhang Y., Wang X., Liu L., Fan Z. (2017). RNA sequencing analysis reveals protective role of kruppel-like factor 3 in colorectal cancer. Oncotarget.

[65] Tang Q., Cang S., Jiao J., Rong W., Xu H., Bi K., Li Q., Liu R. (2020). Integrated study of metabolomics and gut metabolic activity from ulcerative colitis to colorectal cancer: The combined action of disordered gut microbiota and linoleic acid metabolic pathway might fuel cancer. J. Chromatogr. A.

