# Supplementary material for: Maternal Diet Determines Milk Microbiome Composition and Offspring Gut Colonization in Wistar Rats
Source: Nutrients. 2023 Oct 10;15(20):4322. doi: 10.3390/nu15204322 (PMC10609248; doi:10.3390/nu15204322)
Supplement: Supplementary file 1 [file nutrients-15-04322-s001.zip › Supplemetary Table S1.docx]

**Supplementary Table S1.** Relative abundance of the most abundant genera in the milk of Control (C) and Undernourished (U) rats at 4, 14 and 18 days of lactation. ^1^Relative abundance (%) of each bacterial genus is expressed as the median and the interquartile range. ^2^Kruskal-Wallis rank test was used to evaluate differences in relative abundances of major genus between groups (by age and by nutrition type). N = 5-6.

| **MILK** | | | | | | | | | | | | | |
| --- | --- | --- | --- | --- | --- | --- | --- | --- | --- | --- | --- | --- | --- |
|  | CL4 | | UL4 | | CL14 | | UL14 | | CL18 | | UL18 | |  |
| **Phylum**/*Genus* | ***n (%)*** | ***Relative Abundance (%)^1^*** | ***n (%)*** | ***Relative Abundance (%)*** | ***n (%)*** | ***Relative***  ***Abundance (%)*** | ***n (%)*** | ***Relative***  ***Abundance (%)*** | ***n (%)*** | ***Relative***  ***Abundance (%)*** | ***n (%)*** | ***Relative***  ***Abundance (%)*** | ***p-Valor^2^*** |
| **Firmicutes** |  |  |  |  |  |  |  |  |  |  |  |  |  |
| *Lactobacillus* | 6 (100) | 65.93 (21-72.52) | 5 (100) | 72.96 (72.73-86.38) | 6 (100) | 34.53 (18.6-63.05) | 6 (100) | 56.21 (17.26-69.06) | 6 (100) | 37.11 (22.08-39.9) | 6 (100) | 21.83 (9.08-28.84) | 0.037 |
| *Streptococcus* | 6 (100) | 1.61 (1.45-1.87) | 5 (100) | 2.15 (1.62-2.64) | 6 (100) | 4.88 (3.22-15.94) | 6 (100) | 4.22 (3.32-5.71) | 6 (100) | 3.56 (2.25-6.23) | 6 (100) | 2.38 (1.79-2.48) | 0.084 |
| *Romboutsia* | 4 (66.67) | 0.4 (0.09-0.53) | 4 (80) | 0.16 (0.09-0.24) | 5 (83.33) | 0.4 (0.15-0.69) | 4 (66.67) | 0.35 (0.03-0.88) | 6 (100) | 9.6 (4.3-18.82) | 4 (66.67) | 1.2 (0.28-1.89) | 0.0061 |
| *Turicibacter* | 4 (66.67) | 0.51 (0.05-0.98) | 4 (80) | 0.06 (0.03-0.08) | 5 (83.33) | 0.1 (0.05-0.14) | 3 (50) | 0.09 (<0.01-0.19) | 5 (83.33) | 3.6 (1.61-20.96) | 5 (83.33) | 0.94 (0.27-1.47) | 0.069 |
| *Gemella* | 3 (50) | 0.1 (<0.01-0.21) | 5 (100) | 0.25 (0.23-0.26) | 6 (100) | 0.64 (0.41-0.85) | 5 (83.33) | 0.94 (0.52-2.19) | 6 (100) | 0.91 (0.37-1.01) | 6 (100) | 1.79 (1.46-2.41) | 0.00062 |
| *Staphylococcus* | 6 (100) | 2.26 (1.21-2.78) | 5 (100) | 0.22 (0.15-0.34) | 4 (66.67) | 0.16 (0.03-0.21) | 6 (100) | 0.93 (0.73-0.94) | 4 (66.67) | 0.15 (<0.01-0.66) | 6 (100) | 0.49 (0.3-0.6) | 0.003 |
| *Enterococcus* | 2 (33.33) | <0.01 (<0.01-0.05) | 3 (60) | 0.01 (<0.01-0.04) | 2 (33.33) | <0.01 (<0.01-0.03) | 3 (50) | 0.08 (<0.01-2.61) | 1 (16.67) | <0.01 (<0.01-<0.01) | 6 (100) | 1.7 (0.79-1.87) | 0.02 |
| *Clostridium* | 2 (33.33) | <0.01 (<0.01-0.3) | 2 (40) | <0.01 (<0.01-0.04) | 6 (100) | 0.48 (0.25-0.64) | 5 (83.33) | 0.47 (0.27-0.65) | 5 (83.33) | 0.96 (0.36-1.9) | 6 (100) | 0.49 (0.39-0.68) | 0.041 |
| *Globicatella* | 6 (100) | 0.37 (0.26-0.46) | 2 (40) | <0.01 (<0.01-0.17) | 5 (83.33) | 0.81 (0.19-1.23) | 5 (83.33) | 0.45 (0.33-0.75) | 6 (100) | 0.43 (0.32-0.52) | 6 (100) | 0.45 (0.37-0.56) | 0.058 |
| *Veillonella* | 3 (50) | 0.04 (<0.01-0.16) | 3 (60) | 0.46 (<0.01-0.49) | 2 (33.33) | <0.01 (<0.01-0.55) | 2 (33.33) | <0.01 (<0.01-1.49) | 2 (33.33) | <0.01 (<0.01-0.96) | 6 (100) | 0.41 (0.4-0.7) | 0.48 |
| **Proteobacteria** |  |  |  |  |  |  |  |  |  |  |  |  |  |
| *Rodentibacter* | 6 (100) | 10.11 (4.88-11.17) | 5 (100) | 2.32 (0.78-2.48) | 6 (100) | 9.78 (1.25-21.28) | 6 (100) | 4.07 (1.76-9.54) | 6 (100) | 2.69 (1.35-3.77) | 6 (100) | 2.12 (1.91-15.27) | 0.48 |
| *Escherichia.Shigella* | 4 (66.67) | 4.45 (0.02-14.96) | 1 (20) | <0.01 (<0.01-<0.01) | 3 (50) | 0.05 (<0.01-0.28) | 4 (66.67) | 0.15 (0.01-0.47) | 3 (50) | 0.01 (<0.01-0.23) | 4 (66.67) | 0.68 (0.06-1.31) | 0.43 |
| *Alysiella* | 2 (33.33) | <0.01 (<0.01-0.02) | 4 (80) | 0.43 (0.13-0.44) | 2 (33.33) | <0.01 (<0.01-0.92) | 0 (0) | <0.01 (<0.01-<0.01) | 4 (66.67) | 0.43 (0.07-0.64) | 2 (33.33) | <0.01 (<0.01-1.88) | 0.17 |
| **Actinobacteriota** |  |  |  |  |  |  |  |  |  |  |  |  |  |
| *Rothia* | 4 (66.67) | 0.35 (0.08-0.43) | 1 (20) | <0.01 (<0.01-<0.01) | 6 (100) | 1.07 (1.05-2.72) | 6 (100) | 0.91 (0.68-1.86) | 5 (83.33) | 0.62 (0.37-1.14) | 6 (100) | 0.36 (0.3-0.42) | 0.0031 |
| *Corynebacterium* | 5 (83.33) | 0.19 (0.04-0.3) | 3 (60) | 0.17 (<0.01-0.26) | 5 (83.33) | 0.41 (0.14-0.51) | 6 (100) | 0.41 (0.3-0.52) | 4 (66.67) | 0.15 (0.03-0.32) | 3 (50) | 0.04 (<0.01-0.12) | 0.19 |
| **Bacteroidota** |  |  |  |  |  |  |  |  |  |  |  |  |  |
| *Porphyromonas* | 4 (66.67) | 0.1 (0.01-0.25) | 5 (100) | 0.95 (0.67-1.66) | 2 (33.33) | <0.01 (<0.01-0.45) | 1 (16.67) | <0.01 (<0.01-<0.01) | 4 (66.67) | 0.41 (0.08-3.46) | 2 (33.33) | <0.01 (<0.01-3.55) | 0.067 |
| **Cyanobacteria** |  |  |  |  |  |  |  |  |  |  |  |  |  |
| *Aliterella* | 6 (100) | 0.77 (0.51-0.95) | 4 (80) | 0.29 (0.02-0.35) | 2 (33.33) | <0.01 (<0.01-1.29) | 3 (50) | 0.76 (<0.01-3.56) | 2 (33.33) | <0.01 (<0.01-0.01) | 1 (16.67) | <0.01 (<0.01-<0.01) | 0.078 |
| **Fusobacteriota** |  |  |  |  |  |  |  |  |  |  |  |  |  |
| *Fusobacterium* | 2 (33.33) | <0.01 (<0.01-0.07) | 5 (100) | 0.45 (0.44-0.54) | 2 (33.33) | <0.01 (<0.01-0.38) | 0 (0) | <0.01 (<0.01-<0.01) | 4 (66.67) | 0.24 (0.05-1.04) | 2 (33.33) | <0.01 (<0.01-2.78) | 0.047 |
|  |  |  |  |  |  |  |  |  |  |  |  |  |  |
| *Minor_genera* | 6 (100) | 6.36 (2.1-9.2) | 5 (100) | 8.19 (2.79-9.24) | 6 (100) | 0.62 (0.11-8) | 6 (100) | 3.86 (1.84-6.93) | 6 (100) | 2.73 (1.35-7.66) | 6 (100) | 1.83 (1.47-12.1) | 0.84 |
| *Unclassified_genera* | 6 (100) | 4.59 (3.06-7.87) | 5 (100) | 8.85 (8.63-9.38) | 6 (100) | 18.8 (9.14-20.96) | 6 (100) | 7.14 (3.19-55.64) | 6 (100) | 16.83 (9.19-23.26) | 6 (100) | 56.08 (19.45-71.81) | 0.2 |
