# Supplementary material for: Maternal Diet Determines Milk Microbiome Composition and Offspring Gut Colonization in Wistar Rats
Source: Nutrients. 2023 Oct 10;15(20):4322. doi: 10.3390/nu15204322 (PMC10609248; doi:10.3390/nu15204322)
Supplement: Supplementary file 1 [file nutrients-15-04322-s001.zip › Supplemetary Table S2.docx]

**Supplementary Table S2.** Relative abundance of the most abundant genera in the feces of Control (C) and Undernourished (U) offspring rats at 4, 14 and 18 days of lactation. ^1^Relative abundance (%) of each bacterial genus is expressed as the median and the interquartile range. ^2^Kruskal-Wallis rank test was used to evaluate differences in relative abundances of major genus between groups (by age and by nutrition type). N = 5-6.

| **FECES** | | | | | | | | | | | | | |
| --- | --- | --- | --- | --- | --- | --- | --- | --- | --- | --- | --- | --- | --- |
|  | CL4 | | UL4 | | CL14 | | UL14 | | CL18 | | UL18 | | |
| **Phylum**/*Genus* | ***n (%)*** | ***Relative***  ***Abundance (%)^1^*** | ***n (%)*** | ***Relative***  ***Abundance (%)*** | ***n (%)*** | ***Relative***  ***Abundance (%)*** | ***n (%)*** | ***Relative***  ***Abundance (%)*** | ***n (%)*** | ***Relative Abundance (%)*** | ***n (%)*** | ***Relative***  ***Abundance (%)*** | ***p-Valor^2^*** |
| **Firmicutes** |  |  |  |  |  |  |  |  |  |  |  |  |  |
| *Lactobacillus* | 6 (100) | 41.12 (35.85-49.84) | 5 (100) | 88.57 (84.63-90.89) | 6 (100) | 28.93 (15.18-60.74) | 5 (100) | 53.39 (39.49-55.27) | 6 (100) | 41.3 (25.65-44.08) | 6 (100) | 15.3 (10.86-43.29) | 0.016 |
| *Enterococcus* | 3 (50) | 0.13 (<0.01-0.3) | 1 (20) | <0.01 (<0.01-<0.01) | 6 (100) | 8.01 (1.59-10.7) | 5 (100) | 1.97 (0.44-3.95) | 5 (83.33) | 1.43 (0.48-3.99) | 6 (100) | 0.28 (0.21-0.44) | 0.0042 |
| *Streptococcus* | 5 (83.33) | 0.58 (0.11-0.78) | 5 (100) | 2.6 (2.13-2.64) | 6 (100) | 2.43 (1.84-2.91) | 5 (100) | 0.75 (0.4-1.12) | 6 (100) | 1.53 (0.85-2.34) | 6 (100) | 0.47 (0.35-0.67) | 0.0031 |
| *Romboutsia* | 4 (66.67) | 0.23 (<0.01-0.49) | 3 (60) | 0.31 (<0.01-0.4) | 6 (100) | 2.26 (1.25-2.91) | 5 (100) | 1.51 (0.5-2.01) | 6 (100) | 1.63 (0.86-2.22) | 6 (100) | 0.56 (0.3-0.8) | 0.0014 |
| *UCG.005* | 1 (16.67) | <0.01 (<0.01-<0.01) | 0 (0) | <0.01 (<0.01-<0.01) | 6 (100) | 1.9 (0.35-4.09) | 0 (0) | <0.01 (<0.01-<0.01) | 6 (100) | 0.55 (0.35-0.89) | 4 (66.67) | 0.21 (0.04-0.3) | 0.00035 |
| *Eubacterium* | 1 (16.67) | <0.01 (<0.01-<0.01) | 0 (0) | <0.01 (<0.01-<0.01) | 4 (66.67) | 0.48 (0.07-0.92) | 0 (0) | <0.01 (<0.01-<0.01) | 6 (100) | 1.15 (0.74-1.31) | 4 (66.67) | 0.25 (0.04-0.35) | 0.00079 |
| *Veillonella* | 1 (16.67) | <0.01 (<0.01-<0.01) | 1 (20) | <0.01 (<0.01-<0.01) | 2 (33.33) | <0.01 (<0.01-0.62) | 2 (40) | <0.01 (<0.01-0.13) | 2 (33.33) | <0.01 (<0.01-1.11) | 6 (100) | 0.54 (0.4-1.93) | 0.02 |
| *Staphylococcus* | 5 (83.33) | 0.39 (0.26-0.72) | 4 (80) | 0.08 (0.04-0.11) | 4 (66.67) | 0.04 (<0.01-0.09) | 3 (60) | 0.25 (<0.01-0.79) | 2 (33.33) | <0.01 (<0.01-0.04) | 4 (66.67) | 0.13 (0.03-0.28) | 0.1 |
| *Blautia* | 2 (33.33) | <0.01 (<0.01-0.03) | 0 (0) | <0.01 (<0.01-<0.01) | 1 (16.67) | <0.01 (<0.01-<0.01) | 1 (20) | <0.01 (<0.01-<0.01) | 6 (100) | 0.71 (0.37-1.01) | 4 (66.67) | 0.21 (0.05-0.27) | 0.00095 |
| *Colidextribacter* | 1 (16.67) | <0.01 (<0.01-<0.01) | 0 (0) | <0.01 (<0.01-<0.01) | 5 (83.33) | 0.12 (0.07-0.37) | 0 (0) | <0.01 (<0.01-<0.01) | 6 (100) | 0.89 (0.37-1.44) | 5 (83.33) | 0.24 (0.06-0.53) | 0.00022 |
| *Roseburia* | 2 (33.33) | <0.01 (<0.01-0.03) | 0 (0) | <0.01 (<0.01-<0.01) | 3 (50) | 0.1 (<0.01-0.3) | 3 (60) | 0.16 (<0.01-0.3) | 3 (50) | 0.12 (<0.01-0.93) | 4 (66.67) | 0.27 (0.04-0.59) | 0.25 |
| **Proteobacteria** |  |  |  |  |  |  |  |  |  |  |  |  |  |
| *Escherichia.Shigella* | 5 (83.33) | 49.33 (12.24-59.37) | 1 (20) | <0.01 (<0.01-<0.01) | 6 (100) | 29.97 (11.58-38.2) | 5 (100) | 36.74 (15.17-57.6) | 4 (66.67) | 28.6 (4.05-42.17) | 6 (100) | 65 (26.71-73.86) | 0.02 |
| *Rodentibacter* | 5 (83.33) | 1.53 (0.51-1.76) | 4 (80) | 0.85 (0.35-1.79) | 6 (100) | 1.01 (0.94-1.46) | 5 (100) | 1.43 (1.23-5.68) | 6 (100) | 0.22 (0.17-0.48) | 6 (100) | 0.38 (0.2-5.55) | 0.18 |
| **Bacteroidota** |  |  |  |  |  |  |  |  |  |  |  |  |  |
| *Bacteroides* | 3 (50) | 0.01 (<0.01-0.11) | 0 (0) | <0.01 (<0.01-<0.01) | 6 (100) | 3.82 (2.26-4.05) | 3 (60) | 0.56 (<0.01-0.63) | 6 (100) | 2.31 (1.44-3.12) | 6 (100) | 1.37 (0.44-2.84) | 0.00041 |
| *Muribaculaceae* | 1 (16.67) | <0.01 (<0.01-<0.01) | 0 (0) | <0.01 (<0.01-<0.01) | 4 (66.67) | 0.13 (0.03-0.27) | 1 (20) | <0.01 (<0.01-<0.01) | 6 (100) | 3.35 (1.72-5.03) | 4 (66.67) | 1.63 (0.41-2.26) | 0.0012 |
| *Parabacteroides* | 1 (16.67) | <0.01 (<0.01-<0.01) | 0 (0) | <0.01 (<0.01-<0.01) | 4 (66.67) | 0.31 (0.07-0.62) | 2 (40) | <0.01 (<0.01-0.22) | 5 (83.33) | 2.15 (0.32-4) | 2 (33.33) | <0.01 (<0.01-0.11) | 0.017 |
| **Fusobacteriota** |  |  |  |  |  |  |  |  |  |  |  |  |  |
| *Fusobacterium* | 2 (33.33) | <0.01 (<0.01-0.17) | 5 (100) | 0.33 (0.28-0.5) | 2 (33.33) | <0.01 (<0.01-1.16) | 0 (0) | <0.01 (<0.01-<0.01) | 4 (66.67) | 0.22 (0.03-0.68) | 2 (33.33) | <0.01 (<0.01-1.18) | 0.084 |
| **Verrucomicrobiota** |  |  |  |  |  |  |  |  |  |  |  |  |  |
| *Akkermansia* | 1 (16.67) | <0.01 (<0.01-<0.01) | 0 (0) | <0.01 (<0.01-<0.01) | 6 (100) | 0.39 (0.28-0.43) | 0 (0) | <0.01 (<0.01-<0.01) | 4 (66.67) | 0.3 (0.05-1.01) | 1 (16.67) | <0.01 (<0.01-<0.01) | 0.0009 |
|  |  |  |  |  |  |  |  |  |  |  |  |  |  |
| *Minor_genera* | 6 (100) | 1.17 (0.73-2.33) | 5 (100) | 2.12 (1.98-2.6) | 6 (100) | 4.05 (3.36-4.5) | 5 (100) | 1.29 (0.5-1.87) | 6 (100) | 7.67 (2.41-12.36) | 6 (100) | 3.43 (2.9-6.56) | 0.0092 |
| *Unclassified_genera* | 6 (100) | 3.2 (1.34-5.63) | 5 (100) | 3.6 (3.26-6.59) | 6 (100) | 8.05 (4.99-15.79) | 5 (100) | 1.6 (0.39-2.55) | 6 (100) | 11.83 (6.4-16.06) | 6 (100) | 4.23 (3.04-4.36) | 0.024 |
